# Supplementary material for: Job and life satisfaction among emergency physicians: A qualitative study
Source: PLoS One. 2023 Feb 24;18(2):e0279425. doi: 10.1371/journal.pone.0279425 (PMC9955602; doi:10.1371/journal.pone.0279425)
Supplement: S1 Appendix — (DOCX) [file pone.0279425.s001.docx]

***Appendix: Interview guide for physician thriving study***

***DEMOGRAPHICS***

What is your present position? (private practice? Academics?  Pediatrics? Medicine? Family?)

How long have you been in this position?

Age?  Gender? Ethnic group?

All things considered, how satisfied are you with your life?” (0-10)

All things considered, how satisfied are you with your career? (0-10)

How often do you feel burned out from work?

• Never

• A few times a year or less

• Once a month or less

• A few times a month

• Once a week

• A few times a week

• Every day

How often do you feel you have become more callous to people since you took this job?

• Never

• A few times a year or less

• Once a month or less

• A few times a month

• Once a week

• A few times a week

• Every day

***WORK ENVIRONMENT***

Tell me about your job?

What do you like best?

How many hours a week do you work?

What are some of the struggles?

Is there anything about your job that has helped you thrive?

When were you the happiest in your career professionally?  Personally? Why?

When do you struggle the most in your career?  Can you share about that?

What helped the most to have a successful career?

Anything else to add?

***SOCIAL/COMMUNITY ENVIRONMENT***

Tell me about your work-life balance?

Can you share a story about a time when your work-life balance was going well?

What made it go well?

Can you share a story about a time when your work-life balance was not so great?

What made it so?

What helped make your work-life balance successful?

Is there anything else that helped your work life balance?

What would you say were factors that helped you have a successful/happy work-life balance?

What is your approach to money? Are you paid too much, about right, too little? Do you every worry about money? Do you have enough?

***PERSONAL CHARACTERISTICS***

How would you describe yourself as a person?

Introvert? Extrovert? Something else?

What traits do you have that helped you be successful in your job?

What role did friendships play in your career satisfaction?

What role did your family play in your career satisfaction?

Tell me about any core values that you may have?

Can you share about religion or spirituality?  How was that important to you?

Can you share about hobbies or extra activities?  How important were these activities in your career satisfaction?

If there was a resident joining us in this conversation, what advice would you give?

Any other thoughts or insights that we have not covered?

“Thank you for your wisdom and insights, is there anything else you’d like to add before we end?”
