# Supplementary material for: Job and life satisfaction among emergency physicians: A qualitative study
Source: PLoS One. 2023 Feb 24;18(2):e0279425. doi: 10.1371/journal.pone.0279425 (PMC9955602; doi:10.1371/journal.pone.0279425)
Supplement: S2 Appendix — (DOCX) [file pone.0279425.s002.docx]

**Transcribed Interviews**

**JC**

J: What is your present position right now?

Dr: I am both an attending and also a medical director for a couple emergency departments. I oversee a bunch of – four or five different emergency departments.

J: How long have you been in this position?

Dr: 2 years as the regional director

J: All things considered, how satisfied are you with your life, on a scale from 1-10?

Dr: I would say 8

J: And how satisfied are you with your career on scale from 1-10?

Dr: Career? Also 8

J: How often do you feel burned out from work? Never, a few times a year, once a month, once a week, everyday, few times week, few times a month?

Dr: A few times a week, but for a short amount of time

J: Okay, and how often do you feel you have become more callous to people since you took this job?

Dr: I would say, probably once a week. For me, it is a constant thing I am working with. So, I am always reminding myself to be more empathetic. Sorry, I am not going to answer this with a straight answer. So, one of the things you learn in pediatrics is you have to be most disciplined about distancing yourself from the patient because who would ever want to stick a baby with lots of needles and catheters and stuff like that. But on the other hand, you also get tremendous joy from being with kids, so the kind of dichotomy of being the objective callous mind when you need to be versus the cozy friendly, is most pronounced in pediatrics. There is such a compelling need for it so you have to be objective. Emergency meds also lends itself that way because at times you have to be super super callous and objectives because who would want a chest tube shoved in their chest. But then you got to switch to be very careful and empathetic and all that. So, I switch from both those two perspectives all the time. This happens a couple times a day, probably every shift.

J: What do you like best about your job?

Dr: What I like best is at times I have to be clear and objective and analytic but at other times I need to be intuitive, empathetic, and imaginative and it is the fact that I get to do both.

J: What are some of the struggles that you face with your job?

Dr: So, I think that one of the things in our job is that we see lots of horrific things and being in emergency medicine, it makes some of the things last really hard. One of the things is being able to relate those experiences and that mindset to other people and being able to have my job not take away to my family. I want it to contribute rather than take away. I try to make sure to synthesis work life and family life and makes sure they are truly beneficial to each other.

J: When do those struggles occur or when do you struggle the most in your career?

Dr: Its funny, I would say when my schedule opposite from my family’s schedule or when I have to travel a lot.

J: Is there anything about your job that has helped you thrive?

Dr: The intellectual challenge, the challenges, helping people, helping other doctors.

J: You said that one thing that you struggle with is the work-life balance, how do you overcome that? What do you do to not have that struggle?

Dr: Its not the work-life balance, it is more like the work-life synthesis. In other words, its bad when it is work-life balance because it never really works out when you try to balance the two. You always need to make the two kind of interconnect so your life at home informs what you do at work and the values that you experience at home and the experiences you have at work, intertwine and work well with each other.

J: What are factors that have helped you have successful/happy work-life balance?

Dr: I would say, when the home life is exciting and challenging but somewhat organized and when the work life is emotionally meaningful and so they can cross over to each other.

J: So now I am going to ask you about your personal life. How would you describe yourself as a person? Introvert? Extrovert? Or something else?

Dr: It is kind of weird. I think most people view me, when I was young, as an introvert. People nowadays view me as an extrovert. But I think of myself as an introvert – I am introvert on inside and extrovert on the outside. *laughter* that is such an unhelpful answer.

J: Do you think that is a trait that has made you successful at your job?

Dr: Yes, because you need to be both really. The introvert side of you gives you your intellectual and moral integrity while the extrovert side of you allows you to be a leader in the department. Emergency medicine is very much a team sport and if you cannot relate to the nurses, doctors, or patients, and everyone around you, then you end up kind of screwed.

J: That makes sense. What are other traits that have made you successful at your job?

Dr: You need to have a lot of resilience because you get knocked on your butt a lot doing what we do. It’s funny, a [colleague] and I always said that and coined the phrase “Appropriate Arrogance” because who in the fricking world would believe they have the talent or the right to do what we do. So you have to be a little bit full of yourself in order to take the steps to think you can actually do it because in reality, there is no way any sane person would think they can do what we do. Like putting metal things in people’s chest, that is insane. I think part of it is you have to know when to be a little disconnected with reality and have a little fantasy and dream world to aspire to and not to closely examine reality because in reality, there is no way anyone can do what we do. So, having a little fantasy world is part of that. I think being resilient and back bounce on yourself. Having a goal of integrity is very very important because we are told by lawyers and finance people that we have to violate our core and ethical principles all the time, so being able to navigate that is very important. I think that you have to love people but also ideas. You have to do everything *laughter*

J: I am wondering about the role of friendships and the role it plays in career satisfaction.

Dr: It is super important. Yeah friendships are really really really important because both helping out friends and being helped out by friends. It re-energizes you and it gives you perspective and has saved my life many times.

J: What role does your family play in your career satisfaction?

Dr: I think that, with emotional growth, I think that it is really important and you have to be growing all the time at home and in the workplace or else you are screwed – if you are not growing emotionally, it is just not going to work very well. Usually the dog in the family knows I am upset before I do so they provide really good feedback.

J: What are core values you have outside and inside work?

Dr: I think two core values are intellectual integrity that, you know, you have to understand the medicine and what to do medically for the patients. And you can’t compromise that for convenience. The other thing, moral integrity is very important – you need to feel to understand what is happening with your patients and the people around and need to have their moral confidence. And if you think it is all about language or behavior then you miss it completely.

J: Do you have any hobbies or extra activities that you do?

Dr: Yes, I do! I love being in the outdoors and hiking and skiing and also playing squash. I play music and the harmonica. I also like to write and read.

J: And how important are those hobbies in your career satisfaction?

Dr: Huge, very important. In fact, most of my burnout is when career interferes with those hobbies.

J: If there was a resident joining us in the conversation, what advice would you give them?

Dr: One, if you are going to avoid burnout, you need to have hobbies that you are passionate about and you have to have a five-year plan. You need to have a plan about where your career is going. You are challenging yourself and learning and aspiring to something to bring yourself to a better place and you have to have a purpose. You need to know what your purpose is when you walk into the shift. So, it is to see patients or is it to make money or learn, whatever it is, you have to why are there, you have to have a purpose, otherwise you are much more subject to burnout. You need a five-year plan because learning is painful and it hurts and it makes you feel depressed because you don’t know things. So you have to have a reason why you are going through the torture of learning.

J: Are there any other thoughts or insights that we have not covered or anything that you think that matters to your thriving or burnout?

Dr: Because emergency medicine is seeing patients individually, you have to have long term relationships and friendships with your colleague in the real world which provides life’s continuity. On the other hand, if you are too much in the routine of things, you can’t get excited about what is right in front of you. You have to be building a career that you are satisfied with and be satisfied with looking back on it, because emergency medicine is all about in the moment.

J: That is all the questions I have for you. Thank you so much!

**CW**

J: I first want to say thanks for taking the time to talk with me today, I really appreciate it. I want to let you know that this interview will be recorded and then transcribed. This will remain anonymous and names of people or hospitals or programs that were said will be removed. After three months, the recording will be destroyed.

J: What is your present position right now?

Dr: I am an emergency medicine attending and an assistant medical director.

J: How long have you been in that position?

Dr: 1 year

J: How old? Gender? Ethnic group?

Dr: 35, male, Caucasian

J: All things considered, how satisfied are you with your life, on a scale from 1-10?

Dr: I think 7

J: And how satisfied are you with your career on scale from 1-10?

Dr: I think 7

J: How often do you feel burned out from work? Never, a few times a year, once a month, once a week, everyday, few times week, few times a month?

Dr: Probably couple times a month

J: Okay, and how often do you feel you have become more callous to people since you took this job?

Dr: Once a month

J: Tell me about your job, what do you like best about it?

Dr: I like the people I work with and the pathology. The problem solving and for the most part, I really like the patients that I see.

J: How many hours a week do you work?

Dr: For clinical work, I work 12 8 hour shifts a month

J: Is there anything about your job that has helped you thrive?

Dr: No

J: When were you the happiest in your career?

Dr: Probably a 2 week period sometime in the fall of 2018

J: What has helped the most to have a successful career?

Dr: Good mentorship

J: When do you struggle the most in your career and what are those struggles?

Dr: When overwhelmed. I know, when dealing with other obnoxious physicians. That is absolutely the worst.

J: What are the top three cases that haunt you and how have they affected your career?

Dr: A child who passed out and hit his head and I contacted them afterwards and gave the mother my personal cell information and then I got a phone call from the mother that was concerning and then she never picked up the phone ever again. Another was in residency with a complication of a “Trach” that in manipulating it, I locked it temporarily and almost killed someone. For the third one, there was a person who was dying and discussed with two other physicians that he did not want any resuscitations or measures and wanted to die. His daughter felt very differently and she was very upset and screaming at me to do things to help him and was not listening to any kind of explanation and didn’t understand that the patient just wanted to die. That spun out to a long uncomfortable situation and patient complaints. There was no winning scenario there.

J: What did you end up doing in that situation? Did you have to listen to the patient rather than the daughter?

Dr: I did and then an attending went back in and reversed course listening to the daughter temporarily until the son arrived, and with the priest, we were able to deescalate the situation and remove care. I don’t know, ethically, if I could have done the intervention he did to this person.

J: Tell me about your work-life balance?

Dr: I don’t know yet if I have the appropriate balance

J: Can you tell me a story when you think it was going well?

Dr: The other weekend spending time with my phone nowhere near me and turned off. I was completely cut off from work and focusing just on my children.

J: What was a time when it wasn’t working out so well and why?

Dr: Multiple times during the week when I have the nanny scheduled but I am not… I feel like I should be doing something work-wise because I have a nanny but if I am not a work, I should be spending time with my family – and that is a recurring issue.

J: What would or could or did make your work-life balance successful?

Dr: I think more flexibility with child care scheduling and clear hours to avoid spill over and the ability to do other activities with my kids like going on runs with them

J: What is approach to money? Are you about too much? About right? Too little?

Dr: I think about right to too much. About right, but I want to make sure I don’t get caught up in just wanting more

J: Do you ever have to worry about it? And do you have enough?

Dr: Yes and Yes .

J: How would you describe yourself as a person? Introvert? Extrovert?

Dr: I think more of an introvert now.

J: What traits do you have that have made you successful at your job?

Dr: Resilience, perseverance, and inner self-confidence.

J: What role do friendships play in career satisfaction?

Dr: None *laughter*

J: What role does your family play in your career satisfaction?

Dr: 10 – the most.

J: Do you have any core values?

Dr: Focusing on my children or something like that. Making sure that I am present for my kids when I am around.

J: Do you have any hobbies?

Dr: I used to. Climbing, skiing, hiking, camping. Currently, my hobbies are running for my mental health and cooking.

J: If there were a resident joining us in the conversation right now, what advice would you give them?

Dr: Do cool internships and I would say, deliberate, and mindfulness of things that you enjoy about your job.

J: Are there any other thoughts or insights that we have not covered yet?

Dr: Not that I can think of right now.

J: Thanks so much, really appreciate it. Have a good day!

**MG**

J: I first want to say thanks for taking the time to talk with me today, I really appreciate it. I want to let you know that this interview will be recorded and then transcribed. This will remain anonymous and names of people or hospitals or programs that were said will be removed. After three months, the recording will be destroyed.

J: What is your present position right now?

Dr: I am a staff emergency room physician at the [ ] New Mexico hospital.

J: How long have you been in this position?

Dr: Off and on for 19 years. There was a hiatus of about 5 years in there, but for a while

J: Age Gender, ethnic group?

Dr: 65 almost, male, native America

J: All things considered, how satisfied are you with your life, on a scale from 1-10?

Dr: 8 or 9

J: And how satisfied are you with your career on scale from 1-10?

Dr: 7 or 8

J: How often do you feel burned out from work? Never, a few times a year, once a month, once a week, everyday, few times week, few times a month?

Dr: Few times a years

J: Okay, and how often do you feel you have become more callous to people since you took this job?

Dr: Not often

J: Tell me about your job, what do you like best about it?

Dr: I like patient interaction

J: How many hours a week do you work?

Dr: I work 5 24 hour shifts a month

J: Is there anything about your job that has helped you thrive?

Dr: Yeah, I think the ability to have a lot of time when I am not working.

J: When were the happiest in your career, professionally or personally and why?

Dr: I think probably now or this period, certainly the last 5 years.

J: And what has helped you the most to have a successful career?

Dr: Personal growth development and looking for sources of satisfaction in life.

J: When do you struggle the most in your career?

Dr: I think it has been when I have been working in a situation that is not administered well.

J: What are the top three moments or cases in the past that “haunt” you in the past and how have they affected your career?

Dr: At least two out of the three have been unexpected deaths of healthy young people where my interventions were unsuccessful and the person died or got progressively worse. But I think the first one, and I was caring for a sick patient and was working very long hours and was fleeing at the end of a long shift. People wonder how can you work 24 hour shifts but after working 36 hours 24 hours is like nothing. And I remember his wife accosted me as I was running out to my car and she was desperate and I was desperate to leave and that desperate quality of the interaction was there. The sense of reaching the limits of one’s personal abilities and strength and that being an overwhelming experience.

J: Tell me about your work life balance and how do you do that?

Dr: I switch to working in emergency medicine almost 25 years ago and I have almost always worked a part time schedule but with emergency medicine I can sometimes work a full-time schedule but it is never been an average of 40 hours a week. Having a lot more free time for other things really has been key in my career choice. I switched from primary care to emergency medicine and I am very glad I did. Primary care was taking all my time an energy.

J: That seems like you know how to balance you work-life. So, what about a time where your work-life balance not so great and what made it not so great.

Dr: Basically, it was doing primary care. I had thought that is what I intended to do, that was my vision, and I really was not in touch with myself and not aware of what my real abilities were. I had this unrealistic conception of what I was lile and who I wanted to be and it was very much about wanting to be something or feeling like I should be something that I wasn’t. And that got into the way.

J: After switching to emergency medicine, what was it that this career more successful? Was it the more free time?

Dr: I mean I would say that was 90% of it. But also, I think, you know I was able to not take home psychologically and physically, I mean I had to cart this heavy briefcase around with patient files and memos and stuff I had to read. The feeling of coming back to a new shift and not seeing the same patients over and over again with hopeless problems.

J: Seems like you are happy with choice you made. What is your approach to money? Do you think you are paid too much or too little?

Dr: Honestly, I think I am paid too much. However, I am working at 65 because financially I can’t retire, but on the whole money thing, I think people in my situation are over-paid and people have a lot of justification to that and some of them are valid and some of them are not.

J: What are invalid things you think?

Dr: The idea that we work so hard and we have so much reasonability is kind of questionable when you look at other profession like teachers. It’s not true, but it’s kind of how our society has ended up valuing these things. I mean when I say I am paid too much, its relative. I don’t think that we are the only profession that is over-valued.

J: How would you describe yourself as a person? Introvert? Extrovert?

Dr: I think I am fairly balanced. There are periods of my life that I have been more one way than the other. I think in the middle of my life I felt much more extroverted than I was younger, but now during the pandemic, I am quite happy being introverted. My one shift a week is the only interaction I have during the pandemic and that is nice. The spare time to myself is okay and it is definitely balanced.

J: What traits do you have that have made you successful at your job?

Dr: I would say the most important one is sensitivity to other people’s needs and the staff that I work with and being able to kind of respond to those kind of issues that are often unspoken, not overt but getting a feel for what bothers people and what makes them feel happy or validated.

J: What role does friendship play in your career satisfaction?

Dr: I have a couple friends and two of my very good friends are physicians. So friendships, I think that there is something that is supportive about having physician friends who understand things in a different way than other friendships or relatives do.

J: What role does your family play in your career satisfaction?

Dr: I came back to where I am now for family reason so that was defining where I was going to practice. There were a lot of expectations that I had of myself and what other people had of me and what my non-Indian colleagues had of me that made it so difficult One of the great things about transitioning into emergency medicine is that I still see some of those people and they are very happy to see me in the contexts of the emergency department where there is a whole bunch of other people and other doctors and other staff where some of those people used to see me in the clinic. I think the emergency medicine transition made that aspect of family interaction a lot better.

J: Can you tell me a little bit of any core values you have in life or in the workplace.

Dr: You want to have integrity and you want to be responsible. I think you basically want to be self-examined and aware of your biases and intentions. In particular the self-examined part I would stress.

J: Do you have extra hobbies or activities you do outside of work and how do they play into you career satisfaction?

Dr: I kind of have activities that relate to where I live and how I live which is rurally and, on a farm, or ranch. Living remotely and living on the reservation and hunting, growing, you know participating in rituals and ceremonies and those are all kind of the reason I decided to come back to be where I am so that has been a source of satisfaction, I am glad that worked out, as opposed to being a primary care doctor which did not work out.

J: If there was a resident joining us in this conversation, what advice would you give them?

Dr: It would be specific to the person. Don’t go into a primary care, but I really hate saying that but it something that, I guess, just be self-examined and sit with your values and try to be realistic. When I say realistic, I don’t mean holding back your ambitions. When I look at colleagues who are unhappy is that their dissatisfaction has to do with their expectations. Since I went through that with my transition, I am much happier and my colleagues going into something with a bunch of expectations and now feel betrayed. I got out of being betrayed from primary care. The key to avoiding dissatisfaction is being flexible which is to some extent… If you are not sure that you are going to like something or not, don’t make a huge commitment that you can’t change and that usually has to do with money. Don’t get hung up and if it is important for you to go back and do another residency, be able to do that. Have the ability and freedom and what not to do that. Don’t get yourself into a situation that you cannot change.

**DG**

J: I first want to say thanks for taking the time to talk with me today, I really appreciate it. I want to let you know that this interview will be recorded and then transcribed. This will remain anonymous and names of people or hospitals or programs that were said will be removed. After three months, the recording will be destroyed.

J: What is your present position?

Dr: Staff ER doctor and [ ] Hospital

J: How long have you been in this position?

Dr: 3+ years now

J: What is your age?  Gender? Ethnicity?

Dr: 49, male, caucasion

J: All things considered, on a scale from 0-10, how satisfied are you with your life?” (0-10)

Dr: Damn, that’s deep right off the bat. 8

J: All things considered, on a scale from 0-10, how satisfied are you with your career? (0-10)

Dr: I would say 8

J: How often do you feel burned out from work?

Dr: From, work I would a few times a year

J: How often do you feel you have become more callous to people since you took this job?

Dr: Less I would say, actually

J: Tell me about your job?

Dr: I have been in emergency medicine for quite a while, 16 years of clinical emergency medicine. And in that time I have worked at 28 hospitals, I have just worked academic, in small groups, rurally, military, civilian, overseas, and this is honestly the job I think that I have had, at [ ] Hospital. The job here is superb and to me, it is just as good as it can get in emergency medicine. Only concern is the fiscal stability of the place. What do I like best about it? The people are amazing, the nursing culture is really good. My partners are amazing and the people that I work with are great. As far as the MD’s and the mid-levels and the patients are just superb people – they really are some of the best people that I have had the privilege of serving. And that’s a locale dependent thing and the patient population in south San Francisco are great but I am not implying that ER patients everywhere are wonderful but in our little spot they really are.

J: When do you struggle the most in your career?  Can you share about that?

Dr: There are some simple mechanical things – for instance, long strings of single coverage night shifts are challenging and the older I get, the more challenging it gets. That just sort of degrades me physically, mentally, and emotionally and just worn out. Going from nights to days, you feel sort of waisted for two days straight and it takes time to recover. Like anybody else, there is this nagging concern that we might be out of a job, so that creates some stress and doubt and there is one issue that the cost of living in San Francisco is so high so it just challenging. The money I am making here I would be able to buy a four-bedroom house anywhere else but I live in a 1 bedroom here in SF with my girlfriend. That said, I have chosen that intentionally – I am getting the life I wanted based on rational decision. But it is challenging like I don’t have enough put away for retirement and things like that that are long-term stressors and that is due to the immense cost of living. I know that I am very privileged to make what I do and do work that I love in conditions of great respect and authority.

J: Is there anything about your job that has helped you thrive?

Dr: Yes, once again it comes down to the people that I am working with so, especially in the COVID era, I swear to god I have more fun at work than when I am off from work. It is just energizing much of the time. More days than not, I come home from a shift feeling better about life than when I left for it.

J: When were you the happiest in your career professionally?  Personally? Why?

Dr: Happiest in my career I think I would say there are probably 2 high points only looking at my career. One is this job right now and the other was a job that I held in 09’ to 13’ which I was what is called a senior consultant in Auckland, New Zealand. It was at a large hospital and I did have a variety of tasks there – I was the director of disaster management and it was similar to this, I had a great group of colleagues and good patients. They do emergency better is Australia than we do here in AMERICA and they do a better job maintaining a healthy work-life balance and preventing burnout. It is just a great place to live.

J: What are the top three moments or cases that “haunt” you and how they have affected your career?

Dr: Years ago I missed a diagnosis of a spinal epidural abscess and they are hard to diagnose and they were technical problems and the man died three weeks later. It is very “understandable” but it still haunts me because if I had done something different, I could have saved his life. I would say things like when I was working in Iraq, that haunts me in the sense that there is too much trauma all the time and people that I were emotionally close to were involved. A type of case like challenging procedures like an airway or failed airways, thank god I haven’t lost a patient to a failed airway. Its scary because the person will die right away within minutes if you can’t save them. Here is a classic case, a patient came in with horrible tongue angioedema. Her pressure was dropping and heart rate was going down and we couldn’t get IV access - it was a perfect storm of awful shit.

J: Can you share a story about a time when your work-life balance was going well and what made it go well?

Dr: The positive side of work-life balance is literally having the right balance for you it terms of how many shifts and contact hours you work and how many patients per hour. There is a sweet spot for the for every individual person. You have to make sure you have a life and friends but that’s personal. The most poisonous things that can happen is having a bad nursing culture. Some places, it is poisonous and they can make your life hard – “anti-doctor.” It is almost impossible to change that culture if it starts like that. It creates tremendous stress if you have to go into work worrying about who is going to stab you in the back. A super long commute can suck and impact work-life balance even when the job is not bad.

J: Can you share a story about a time when your work-life balance was not so great?

Dr: There are certain things you do as an ER doctor that just degrade you – when it feels like the management above you is just using you to make money. If you have the feeling your work is contingent like you could get laid off any minute – those factors degrade you and make you feel shitty over time. Also, just too much physiological stress.

J: How would you describe yourself as a person? Introvert? Extrovert? Something else?

Dr: Everyone will tell you I am an extreme extrovert but that is complete bullshit because I am actually an introvert who has been faking it as an extrovert. Most of us are that way.

J: What traits do you have that helped you be successful in your job?

Dr: I have a biased towards action. If you can get into a situation and do something immediately, I just delve right in. I don’t pause, I take that first step right away. If there is a fire, I put it out right away. I am very inquisitive – I am always thinking about how I can improve – kind of like a review cycle. Do contemplate, do again.

J: What role do friendships play in your career satisfaction?

Dr: The most important thing! If you lose all your money, you can always make more money. But you can’t do that with human relationships are the most important things. I like the job for many other reasons but friendship. I don’t know if I have confidence in the corporations, per se, but I have confidence in my friendship. They are the most important thing

J: Tell me about any core values that you may have?

Dr: Being industrious, like not lax, trying to accomplish your mission everyday. Just humanism and it sounds old school but being able to comfort people. It is required to have physical courage and be a little bit bold when facing aggressive assholes. Having the drive to jump into the fight and finish what you started in addition – perseverance. It took practice and cultivation but I think that is part of what I am.

J: Can you share about hobbies or extra activities?

Dr: Yes I do. For one thing, I have done martial arts – combative. I have trained groups of people in the same thing. Teaching civilian groups how to prepare for natural disasters, building teams like that and instructing other people has been an ongoing hobby for me and putting in hundreds of hours over the last several years. I am nerd, I like to chill and read a lot, and basic athletics stuff like swimming and going to the gym. Most of my hobbies have gone away during COVID which is a shame. I like nature too. Another thing, I have a 3-year-old so I spend a lot of time with her.

J: How important were these activities in your career satisfaction?

Dr: I don’t know, mid-range. I would put in at a 5 if it was on a scale from 1-10. If I lost the hobbies but still had the job and friends, I would be fine.

J: If there was a resident joining us in this conversation, what advice would you give them?

Dr: One, I would say, you are a resident, and you are training right now and you should make it hard on yourself, not easy. We are looking for someone that is trustworthy more than someone who is a straight A student because we are all straight A students. We care about that you are going to be durable and you will be cheerful and take great care of your patients.

J: Any other thoughts or insights that we have not covered or anything else you can think of that matters to your thriving?

Dr: It is something that is so huge and neither you nor I can realty do anything about it. Right now there is this titanic of civilization values going on in American and much throughout all of the west. Are we focused on humans and improving and supporting humans, or are we only caring about money. The way that that plays out in emergency medicine, we are told two things at once: on one hand you are a valued employee and we want to help you grow in your career and on the other hand, you are a cog designed to make revenue and have to optimize you to make the most revenue. It is inconceivable that these two values will not clash all the time. Which direction are going in as a society? Are we going in a direction where human workers are valued only in terms of what their return on investment is to the capital class or are we heading in a direction where we truly are humane people and optimizing humanity is our main goal. We as a society have not decided on that yet. It is such a huge macro scale thing.

**SS**

J: I first want to say thanks for taking the time to talk with me today, I really appreciate it. I want to let you know that this interview will be recorded and then transcribed. This will remain anonymous and names of people or hospitals or programs that were said will be removed. After three months, the recording will be destroyed.

J: What is your present position?

Dr: Clinical Physician and I work at two hospitals. They are both community hospitals in the Bay Area.

J: How long have you been in this position?

Dr: I moved to the bay area in September of 2019 so almost a year.

J: What is your age?  Gender? Ethnicity?

Dr: 32, female, asian

J: All things considered, on a scale from 0-10, how satisfied are you with your life?” (0-10)

Dr: 8, pretty satisfied

J: All things considered, on a scale from 0-10, how satisfied are you with your career? (0-10)

Dr: 8

J: How often do you feel burned out from work?

Dr: once a month

J: How often do you feel you have become more callous to people since you took this job?

Dr: often

J: Tell me about your job? What do you like best?

Dr: I like the diversity in terms of patients and cases that I get. The variety it terms of what you see you never get bored and you are always surprised by your patients and you humbled. It is a humbling job. You go to school and learn all of these and people still surprise you. You know things don’t go by the book so recognizing that and being humbled by that is something to go in every shift with that attitude.

J: When do you struggle the most in your career?  Can you share about that?

Dr: My career has not been that very long. But I think so far is going through residency and training. The challenging that come with that. Haven’t had the years to get burnt out yet.

J: Is there anything about your job that has helped you thrive?

Dr: I think having a good working relationship with people you are working with. Nurses, everyone around you and it really helps. Treating every patient like a family member. If you go into the shift with that attitude, you will have a more pleasant shift.

J: What are the top three moments or cases that “haunt” you and how they have affected your career?

Dr: Most of them have been in residency but I had one woman who came in and was Saudi Arabian and did not speak any English and this was in my second year in residency. She had back pain and had no other symptoms and screaming in the halls. She came back later the next day and ended up dying in the ICU. Another one was a guy who came in with abdominal pain and he looked great and sent home and he ended up coming back and being super sick and had heart failure. He had dead bowel from his heart. This guy came in dead, and they brought in a dead body and you are trying to get a line and the smell of that and I still can’t eat anything that burnt. So mostly missed cases and things having to deal with and people dying in horrible ways, a little bit of PTSD from that.

J: Tell me about your work-life balance and what you do to maintain a healthy one?

Dr: I think it is much easier in the ER than in other professions and that is one of the main reasons I chose the ER. Shift work, you can choose the number of shifts that you work. Right now with COVID it is different but usually I take off time every month to travel somewhere. Being able to be active and exercise and cooking is very important. Having a healthy family life and that social life is really crucial.

J: Can you share a story about a time when your work-life balance was not so great? What made it so?

Dr: Residency. Post residency life has been pretty great so far. I mean I get a little tired and working odd hours and you have just weird shifts. It becomes challenging when your partners schedule is different than your

J: What is your approach to money? Are you paid too much, about right, too little? Do you every worry about money? Do you have enough?

Dr: I think we are paid very fairly for what we do. Of course everyone wants to be paid more. We get paid pretty well. I mean could we get paid more? Sure. But would it make me happier? No, probably not, maybe temporarily.

J: How would you describe yourself as a person? Introvert? Extrovert? Something else?

Dr: I think I am an extrovert in small groups but a little bit of social anxiety after that.

J: What traits do you have that helped you be successful in your job?

Dr: I think having a good working relationship with people like nurses and being a nice person – not being an asshole. That would help a lot of people, don’t be an asshole. I try not to be an asshole but sometimes I am.

J: What role do friendships play in your career satisfaction?

Dr: I think you make really good strong friends during residency and these are your friends and colleagues and when you have a bad day, you can text them and call them. Knowing that people are there and they have your back. And it is exciting cuz you can always talk to someone. It plays a big role I would think

J: What role do your family play in your career satisfaction?

Dr: My husband is an anesthesiologist so we can talk about similar things and different things. There is overlap, so overall I am pretty pleased and isn’t boring that were are both doctors.

J: Tell me about any core values that you may have?

Dr: I think treating humanity as humanity and understanding that everyone has a different story. And people come to you and have to judge them in those couple minutes but you are only seeing a small slice of their life and that there is a lot more to it than just that. They have there own story. You shouldn’t forget that.

J: Can you share about hobbies or extra activities?  How important were these activities in your career satisfaction?

Dr: I like rock climbing and diving. If you going into the ER, you have to pick up some sports. Everyone is out-doorsy so I have become more out-doorsy. I think it is important to have these hobbies because it helps maintain a certain amount of sanity when you are seeing things that are difficult.

J: If there was a resident joining us in this conversation, what advice would you give them?

Dr: I think just treat your patients like your family, that will help you with your career satisfaction and help you from being burnt out.

**SM**

J: I first want to say thanks for taking the time to talk with me today, I really appreciate it. I want to let you know that this interview will be recorded and then transcribed. This will remain anonymous and names of people or hospitals or programs that were said will be removed. After three months, the recording will be destroyed.

J: What is your present position?

Dr: I am a staff ER physician at Seton Medical Center

J: How long have you been in this position?

Dr: 2 years

J: What is your age?  Gender? Ethnicity?

Dr: 37, male, Caucasian

J: All things considered, on a scale from 0-10, how satisfied are you with your life?” (0-10)

Dr: I would say 9

J: All things considered, on a scale from 0-10, how satisfied are you with your career? (0-10)

Dr: 8

J: How often do you feel burned out from work?

Dr: Few times a year

J: How often do you feel you have become more callous to people since you took this job?

Dr: few times a year

J: Tell me about your job? What do you like best?

Dr: I like the people that I work with and feeling that I look forward to seeing the people that I work with and we work collaboratively to do something that matters to people and to provide good care and that we are all working towards a similar goal. It is a supportive environment

J: When do you struggle the most in your career?  Can you share about that?

Dr: What it is I want to be doing? Is working as an ER doc what I want? Is it something else? What do I want my career to look like? I like my job but I want to do more global work and I don’t know if that is in healthcare or something else related to healthcare. I struggle cuz I like my job but if there is something else.

J: Is there anything about your job that has helped you thrive?

Dr: Having management and a boss that you feel supported by and challenges you to improve and to learn new things and sort of take on certain responsibilities. Some of my bosses have cared about me as a person more than just a worker. Having good management and superiors that are good mentors.

J: What are the top three moments or cases that “haunt” you and how they have affected your career?

Dr: During residency I had a patient who got hit by a car crossing the street that is a street that I cross all the time. He ended up dying due to brain injury. He was a friend of a friend so that stuck with me cuz it so could have been me. Also, I had a two-year-old kid with head symptoms and he looked well and he had a normal exam so we didn’t scan him and then he came back the next day due to cardiac arrest which was caused by a brain tumor. I had a case on the Alaskan cruise and an elderly patient had a GI bleed and it wouldn’t stop. And the coast guard had to come and pick him up on the helicopter.

J: Tell me about your work-life balance?

Dr: COVID has thrown a wrench into that. I am involved in playing tennis, and all sorts of sports and that has been a huge balance playing all those sports. All of it has been cancelled but I have good friends across the country that I travel and see and my partner and I go on vacations a lot. I am glad I have had work during COVID and it has given me a lot more social balance. I’d like to do some volunteer but I move around a lot so I am still trying to make everything else balance well.

J: Can you share a story about a time when your work-life balance was not so great?

Dr: During residency when you are working so many hours. I was motivated a lot by money so I would pick up a lot of shifts and was very happy with my life. It is easy to get caught up working as an ER doc to make money. Trying to not have money be the focus of me working is a good way to maintain balance bet.

J: What is your approach to money? Are you paid too much, about right, too little? Do you every worry about money? Do you have enough?

Dr: Paid about right. It is not an easy job – the night shifts and weekends – the fact going into medicine causes so much debt. The amount we get paid with what fits with the job and what is required.

J: How would you describe yourself as a person? Introvert? Extrovert? Something else?

Dr: Somewhere in between. I like meeting people but I like to just read and learning languages on my own. I notice when I am being to much of one than the other.

J: What traits do you have that helped you be successful in your job?

Dr: Obsessive about things. In terms of training to become a doctor – studying to know as much as I can and the way I work. I like things flow properly and patients aren’t staying longer than they need to and tests are being done. I am sociable with people and get along with everyone. I see things through and I don’t like to quit things.

J: What role do friendships play in your career satisfaction?

Dr: So I like having a job that is sort of like a stable job where I know the people and the hospital system. You manage an emergency room more efficiently when everyone gets a long with each other. Makes it more enjoyable to be friends with the people and be a little social with the other doctors.

J: What role do your family play in your career satisfaction?

Dr: I have a long term boyfriend and we have moved around a lot and he has changed careers. It has been really helpful for someone to do that with and my parents are also very supportive in my becoming a doctor. They want me to not work so much or a lot

J: Can you share about hobbies or extra activities?  How important were these activities in your career satisfaction?

Dr: It is super important. I would be unsatisfied if I couldn’t do my activities. If I had to work so much where I couldn’t do those I would be unhappy.

J: If there was a resident joining us in this conversation, what advice would you give them?

Dr: Pick a place where you want to live not the place that pays you the most. Figure out the job and money later. Figure out what you like besides medicine.

**MI**

J: I first want to say thanks for taking the time to talk with me today, I really appreciate it. I want to let you know that this interview will be recorded and then transcribed. This will remain anonymous and names of people or hospitals or programs that were said will be removed. After three months, the recording will be destroyed.

J: What is your present position?

Dr: Emergency medicine attending

J: How long have you been in this position?

Dr: 1 year

J: What is your age?  Gender? Ethnicity?

Dr: 31, female, Filipino.

J: All things considered, on a scale from 0-10, how satisfied are you with your life?” (0-10)

Dr: 6

J: All things considered, on a scale from 0-10, how satisfied are you with your career? (0-10)

Dr: 6

J: How often do you feel burned out from work?

Dr: few times a month

J: How often do you feel you have become more callous to people since you took this job?

Dr: maybe once a month

J: Tell me about your job? What do you like best?

Dr: I like the flexibility and I really like the shift work.

J: When do you struggle the most in your career?  Can you share about that?

Dr: When you are taking care of a younger patient and think back to other outcomes with other young patients and think about how you could have done something different. Also when you don’t feel supported in the work place either by your staff or consultants.

J: Is there anything about your job that has helped you thrive?

Dr: Having really good support. My partner is amazing and is there for me all the time. Your boss having your back is amazing.

J: What are the top three moments or cases that “haunt” you and how they have affected your career?

Dr: One of those where it is a little kid. It happened at the beginning of my shift and it is hard because you try to bury it down and not think about it. Having someone die under your watch is not something a lot of people experience. There was a case where a consultant needed to hear the advice from someone else.

J: Tell me about your work-life balance?

Dr: I like have a few shifts and then a bunch of days off in between. In residency my boss said it is like candy but amazing in small batches. Lots of days off in the middle of shifts helps to refresh.

J: What is your approach to money? Are you paid too much, about right, too little? Do you every worry about money? Do you have enough?

Dr: I have a lot of loans so nothing is too much right now.

J: How would you describe yourself as a person? Introvert? Extrovert? Something else?

Dr: Introvert. I love people but one person at a time.

J: What traits do you have that helped you be successful in your job?

Dr: I do enjoy learning about people. Being able to relate to the patients and treating your patients like family. It helps, I think.

J: What role do friendships play in your career satisfaction?

Dr: They are so important. I am still very close with my residency class and we talk all the time.

J: What role do your family play in your career satisfaction?

Dr: My parents were just happy I graduated med school.

J: Tell me about any core values that you may have?

Dr: General happiness over ambitions. If I have to sacrifice family time to get higher up, I will not do that. Value relationships a lot.

J: Can you share about hobbies or extra activities?  How important were these activities in your career satisfaction?

Dr: I like to relax And listen to podcasts. These activities are very important because I need a break from work!

J: If there was a resident joining us in this conversation, what advice would you give them?

Dr: Know what your priorities are and don’t forget them. Sometimes you get lost in the resident life of work-sleep-work-sleep.

**JT**

J: I first want to say thanks for taking the time to talk with me today, I really appreciate it. I want to let you know that this interview will be recorded and then transcribed. This will remain anonymous and names of people or hospitals or programs that were said will be removed. After three months, the recording will be destroyed.

J: What is your present position?

Dr: Emergencey physician and directing of emergency services for EMA and emergency room director at shasta medical center.

J: How long have you been in this position?

Dr: 8

J: What is your age?  Gender? Ethnicity?

Dr: 54, male, white

J: All things considered, on a scale from 0-10, how satisfied are you with your life?” (0-10)

Dr: 7

J: All things considered, on a scale from 0-10, how satisfied are you with your career? (0-10)

Dr: 9

J: How often do you feel burned out from work?

Dr: super rare

J: How often do you feel you have become more callous to people since you took this job?

Dr: at times, but pretty rare, few times a month

J: Tell me about your job? What do you like best?

Dr: I enjoy working and teaching with the mid levels that we provide. And I enjoy making improvement in the efficientcy and the projects that I initiate.

J: When do you struggle the most in your career?  Can you share about that? What are some of the struggles?

Dr: I struggle with a certain type of provider that I struggle with giving perhaps negative feedback and what I notice that those provide are always people traveling in and highly recommended by y bosses but just not good for the department.

J: Is there anything about your job that has helped you thrive?

Dr: Having positive feedback from either my CEO or my superiors.

J: What helped the most to have a successful career?

Dr: Having supportive superiors.

J: What are the top three moments or cases that “haunt” you and how they have affected your career?

Dr: One case was a guy in his late 40s who had a family came in with [] and I gave him 20 of cartisan and he flatlined and then went pulseless. I have never given that much cartisan anymore. Another one, a gunshot wound to the chest and we are trauma room. He lost his pulse on the way and then we cracked his chest and two weeks later he walked out of the hospital. That was a great success story.

J: Tell me about your work-life balance?

Dr: I don’t have a healthy one, I probably work more than most people and I enjoy my work and the other reason is for financial reasons. I was taking each month, two to three days, to spend with just my family. I would force myself to go somewhere with my family.

J: Can you share a story about a time when your work-life balance was not so great?

Dr: Right now it is not good because my wife is ill. I am working a lot more right now and things are disrupted at home because of her illness. Right now, for the last 8 months my work-life balance is not as good – its complicated.

J: What is your approach to money? Are you paid too much, about right, too little? Do you every worry about money? Do you have enough?

Dr: About right. From a clinical stand point, I think it should be a little better than what it si, but I can’t complain too much when you look at all the other people in this country.

J: How would you describe yourself as a person? Introvert? Extrovert? Something else?

Dr: I am an extrovert.

J: What traits do you have that helped you be successful in your job?

Dr: I am perseverant and that is my strongest traits. Once I set a goal I work towards it until it is reached. Initially in my career as a director, I lead with an iron fist and that helped me at that time in my career because, one I was young, and at the same time I walked the walk and talked the talk. I wouldn’t ask anything I wouldn’t do my self – one of my strongest points being in a leadership position is leading by example.

J: What role do friendships play in your career satisfaction?

Dr: Very high. I enjoy working with all my providers and think of them as friends. I never wanted to be friends with those I oversaw and I thought it would be difficult and sticky situation. I first isolated myself from them and this is the longest I have been at one hospital and the longest director at one site. It was investable to become friends with the people that I work with. It has helped my ability to do my job and lead them and ask them things that need to be done for the department.

J: Tell me about any core values that you may have?

Dr: #1 core value is callous and jaded by the drugs and the alcohol and the abuse but at the same time we may criticize or make judgement of that but I always treat people that way I want to be treated.

J: Can you share about religion or spirituality?  I want to know about the things inside of you that helps you thrive. How was that important to you?

Dr: I am spiritual though I haven’t been going to church. I started to do that a year ago but because of COVID but I hope to get back to that. I am very spiritual in the sense that I always feel god will provide that to me that whatever it is I need. It might not be the path that I want or like but everything will be okay. If I hadn’t had that belief I would have jumped off a cliff 20 years ago. I am very faithful in the belief that god is there for me and support me and it is not uncommon that I will pray before my shift and he will provide me with whatever I need t provide the best of care to my patients that day.

J: Can you share about hobbies or extra activities?  How important were these activities in your career satisfaction?

Dr: I work so much so I can do the things that I like to do. Fish, hunt, exercise and just purchased a home so I like to garden and I have an 11 year old daughter and teaching her how to grow food. Very important for career satisfaction.

J: If there was a resident joining us in this conversation, what advice would you give them?

Dr: I have never been good a work life balance so I think people in the younger generation are better than that and have a much better balance.

J: Any other thoughts or insights that we have not covered or anything else you can think of that matters to your thriving/burnout?

Dr: I guess nursing. We have good and great nurses that are fun to work with so having support at work is important and being able to work together. The ability to work together without fighting or without malleus is very important.

**MA**

J: I first want to say thanks for taking the time to talk with me today, I really appreciate it. I want to let you know that this interview will be recorded and then transcribed. This will remain anonymous and names of people or hospitals or programs that were said will be removed. After three months, the recording will be destroyed.

J: What is your present position?

Dr: Staff Physician at shasta region medical center in redding CA

J: How long have you been in this position?

Dr: 23 years

J: What is your age?  Gender? Ethnicity?

Dr: White, 56, Female

J: All things considered, on a scale from 0-10, how satisfied are you with your life?” (0-10)

Dr: 8

J: All things considered, on a scale from 0-10, how satisfied are you with your career? (0-10)

Dr: 8

J: How often do you feel burned out from work?

Dr: couple of times a year

J: How often do you feel you have become more callous to people since you took this job?

Dr: All the time

J: Tell me about your job? What do you like best?

Dr: I like that is shift work and it is predictable. I like the social aspect of it and different people each time. I like the variety of it. I like the lack of continuity of care – I would go nuts if I see the same people day in and day out.

J: How many hours a week do you work?

Dr:

J: When do you struggle the most in your career?  Can you share about that? What are some of the struggles?

Dr: I think the struggles come when you are first graduated from med school and choosing what field you want to go into.

J: Is there anything about your job that has helped you thrive?

Dr: I think probably the flexibility with the job. I can tell my family when I am going to be home

J: What are the top three moments or cases that “haunt” you and how they have affected your career?

Dr: There are always cases that stand out. I tend to pop off at work a lot and cuss and hang up the phone and bitch about something. For me personally, when I leave the hospital, I leave it behind. Although, I still have cases that haunt me. A fourth month baby came in and it was the day before Christmas and the baby died and when I went in to talk to the family and they all screamed at me saying that I killed their child. I never wanted to work the day before Christmas again because I didn’t want to jinx myself. That’s weird cuz I am not a superstitious person. I have only been sued once and that case has stayed with me for as along as it took. And that has stuck with me because it was hard to go to work because you feel violated and don’t want to talk to patients anymore and think you will get sued for anything. I just lost my confidence.

J: Tell me about your work-life balance?

Dr: When I started at this job and my boss told me that I should only work 12 shifts a month. He told me “Here is the deal, every emergency room physician has a set number of shifts in them. You can work like a dog and use them and burnout early. Or you can spread out your work so you don’t burnout. The key for me is not working a lot of shifts. Everyone works that out for themselves. If you want more money work more but if you don’t need to, work less. I would rather have my vacations scattered in short amount of times, I need a bunch of days off. I can’t work too many shifts in a row.

J: How would you describe yourself as a person? Introvert? Extrovert? Something else?

Dr: I am pretty extroverted. I am pretty social and irreverent. I am known for my potty mouth and saying what I think. I tend to be brash and say what I think and get it off my chest. I don’t keep a lot inside and people know me for that. That is what keeps me sane. I am not an administrator and so I don’t worry about the big things I just do what I am paid for. I have seen a lot of administrators come and go throughout this hospital.

J: Do you have a healthy nursing culture?

Dr: Yes and it is so important to have a healthy one because then everyone works well with each other. I am well like by the staff so that makes my job a little easier. If the nurses are unhappy it wont be a good.

J: Can you share about religion or spirituality?  I want to know about the things inside of you that helps you thrive. How was that important to you?

Dr: I am not deep person or spiritual person. There is not much inside of me that guides. I didn’t go into medicine to help people, I thought it was intellectually challenging.

J: Can you share about hobbies or extra activities?  How important were these activities in your career satisfaction?

Dr: Sometimes I feel like I don’t. I cook and travel to Europe whenever we can. We live in a place that is very outdoorsy so I do dirt biking and skiing and boating and exercise.

J: If there was a resident joining us in this conversation, what advice would you give them?

Dr: I would probably tell them have a sane career. Don’t work yourself to death. Have a great rapport with you co-workers and your life will become more painful if you don’t have a great relationship with your coworkers. Don’t live a lifestyle that is like a ball and chain around your neck. Live comfortably within your means.

J: Any other thoughts or insights that we have not covered or anything else you can think of that matters to your thriving/burnout?

Dr:

**PS**

J: I first want to say thanks for taking the time to talk with me today, I really appreciate it. I want to let you know that this interview will be recorded and then transcribed. This will remain anonymous and names of people or hospitals or programs that were said will be removed. After three months, the recording will be destroyed.

J: What is your present position?

Dr: Emergency medicine physician and I work at shasta regional medical center.

J: How long have you been in this position?

Dr: Almost 2 years

J: What is your age?  Gender? Ethnicity?

Dr: 41, white, male

J: All things considered, on a scale from 0-10, how satisfied are you with your life?” (0-10)

Dr: 10

J: All things considered, on a scale from 0-10, how satisfied are you with your career? (0-10)?

Dr: 10

Dr: 10. If I had to do everything all over again, I would do exactly the same thing. I had a business degree and then went into medicine. I valued the experience and everything I have had but I feel like there is a lot now and I am working for a business and someone who profits off of me and there is not a lot of hope of climbing the corporate ladder.

J: How often do you feel burned out from work?

Dr: Every time I go to work.

J: How often do you feel you have become more callous to people since you took this job?

Dr: Every day

J: Tell me about your job? What do you like best?

Dr: I like working with different people and seeing different patients and people with different backgrounds and I like the medicine and all that stuff.

J: When do you struggle the most in your career?  Can you share about that?

Dr: Post night shifts. I used to work 15-18 shifts a month but just recently transition to 12 a month and my goal is 0 times a month.

J: Is there anything about your job that has helped you thrive?

Dr: Relationships at work. The people I work with are amazing and I love working with the people that I am around.

J: Tell me about your work-life balance?

Dr: I have 5 kids and when you have that many kids you can’t have a work-life balance. Shift work is difficult. But the advantage is when you are off, you are off.

J: What is your approach to money? Are you paid too much, about right, too little? Do you every worry about money? Do you have enough?

Dr: Too little. I don’t have to worry about money now but if I were to continue down the course I am going I will have to.

J: How would you describe yourself as a person? Introvert? Extrovert? Something else?

Dr: I am an introverted-extroverted

J: What traits do you have that helped you be successful in your job?

Dr: The introversion helps you to study and do well, and the extroversion helps you be good in a team. Work ethic is a trait that has made me successful.

J: What role do friendships play in your career satisfaction?

Dr: Emergency medicine is you go and work your shift and you are done. There is as little or as much friendship that you want because you can show up to work and leave.

J: What role do your family play in your career satisfaction?

Dr: Big part of why I went into emergency medicine.

J: Can you share about religion or spirituality?  I want to know about the things inside of you that helps you thrive. How was that important to you?

Dr: I would have answered that differently 10 years ago. For spirituality, but I think we are all apart of the human race and if you apart of something that is not inclusive that your view may be skewed. I enjoy taking care of people of all different walks of life and problems and ages and belief systems and orientation. I like the fact that anyone can walk in and I can see them. I think you can love every single person in the world as a human being regardless of what you do

J: Can you share about hobbies or extra activities?  How important were these activities in your career satisfaction?

Dr: I play golf and exercise and take my kids to do stuff outdoors. I also stock market stuff and trade options. I made a lot of money recently and that is my gateway. I would say these are moderately important but if you don’t like what you do at work you won’t like what you do outside of work.

J: If there was a resident joining us in this conversation, what advice would you give them?

Dr: Think outside the box and don’t be afraid to start your own company. Don’t think just about medicine. I would expand your horizons outside of medicine.

J: Any other thoughts or insights that we have not covered or anything else you can think of that matters to your thriving/burnout?

Dr: No. I am young for my career but I am ready to leave medicine.

**PK**

J: I first want to say thanks for taking the time to talk with me today, I really appreciate it. I want to let you know that this interview will be recorded and then transcribed. This will remain anonymous and names of people or hospitals or programs that were said will be removed. After three months, the recording will be destroyed.

J: What is your present position?

Dr: Medical director of the emergency department at

J: How long have you been in this position?

Dr: 6 and a half years

J: What is your age?  Gender? Ethnicity?

Dr: 50, male, India.

J: All things considered, on a scale from 0-10, how satisfied are you with your life?” (0-10)

Dr: 10

J: All things considered, on a scale from 0-10, how satisfied are you with your career? (0-10)

Dr: 10

J: How often do you feel burned out from work?

Dr: few times a year

J: How often do you feel you have become more callous to people since you took this job?

Dr: opposite, a father figure.

J: Tell me about your job? What do you like best?

Dr: Everyday there is a different scenario or theme and layers of excitement. Its like a birthday present every day of your life. It’s a total surprise and there are not many careers in the world where you have such a tremendous impact on someone life when time is of the essence.

J: How many hours a week do you work?

Dr:

J: When do you struggle the most in your career?  Can you share about that?

Dr: I think a lot of people who have kids and family and investments and parents who are getting sick, we struggle when everything seems to be hitting the fam at the same time. Your work is stressful and there is a financial loss. For most of us it occurs in the same month.

J: Is there anything about your job that has helped you thrive?

Dr: We tend to make mistakes when stress is not there. I thrive when everything is falling apart and I am stressed out and overwhelmed.

J: What helped the most to have a successful career?

Dr: Family, my wife, having a good relationship and working hard on your relationship is one of the most important things for me. The other thing sis being nice to people around you.

J: What are the top three moments or cases that “haunt” you and how they have affected your career?

Dr: Three babies all under three months and sudden infant death syndrome. I saw all three and had to tell all three mothers that their babies have died. I took a couple of weeks before I fell apart and broke down because you try to come back to work and not show your weaknesses, but the longer you put that wall up the harder you fall.

J: Tell me about your work-life balance?

Dr: I try to have as much fun and keep the little kid in myself as much as possible. I love hanging out with my friends and cooking in the kitchen with my wife. This is an opportunity to go out and do this and getting away from work. Keeping yourself occupied with different things and not being lazy.

J: Can you share a story about a time when your work-life balance was not so great?

Dr: When I came out of residency and lots of people have a huge loan debt and I just got married. Being in debt is something I did not want so for a couple of years I worked every day of my life. I burned out and wasn’t enjoying life and I hit a wall. I wasn’t getting much time to exercise and eating right and how relationship was struggling so it was affecting my attitude at work.

J: What is your approach to money? Are you paid too much, about right, too little? Do you every worry about money? Do you have enough?

Dr: Paid just about right. You know its funny there is a certain amount of money that you make and then after that you can’t define happiness after that, you are probably not spending your money right. At some point, life will be the same no matter how much you get paid. I don’t have to worry about money, I don’t need some of the things that other people need like big nice cars. I need food on the table and I don’t want debt. Just to be afloat is enough for me. I was rich once I paid off my loans.

J: How would you describe yourself as a person? Introvert? Extrovert? Something else?

Dr: I am socially an extrovert but I am introverted when I don’t know you, I am a private person. Its not that I am not trusting, it is just I am careful

J: What traits do you have that helped you be successful in your job?

Dr: You can’t work in the ER if you are not a team player so extroversion is very important. You need to be somewhat of a call maker to thrive in the emergency department. Just having passion and stamina is important to be successful. You need to have stamina, it is very important.

J: What role do friendships play in your career satisfaction?

Dr: They are very important. Who is going to help you when you fall? You need to have a good social support. It is not if it is coming, it is when it is coming.

J: Tell me about any core values that you may have?

Dr: First and foremost is your morals and how you were raised and how you treat people. That starts at a young age and the way you treat people around you. If you treat everyone well, you will stand out in a crowd.

J: Can you share about religion or spirituality?  I want to know about the things inside of you that helps you thrive. How was that important to you?

Dr: I am Indian and Hindu, but I am not the most religious person in the world. I respect every religion out there and I think it is important to respect everyone. I am kind of a spiritual person and karma person. Part of the reason I am nice is because what goes around come around.

J: Can you share about hobbies or extra activities?  How important were these activities in your career satisfaction?

Dr: I like watching tv. I like going out to eat and wine tasting and traveling. I like seeing different cultures and different parts of the world. They are very important cuz you gotta have fun outside of work.

J: If there was a resident joining us in this conversation, what advice would you give them?

Dr: Keep your mind free, you are a young soul and this is stressful time in your career but it’s the most beautiful point of career because your growth spurt and learning peaks will be at your best. You are so hungry for knowledge. Build as many good relationships as you can.

J: Any other thoughts or insights that we have not covered or anything else you can think of that matters to your thriving/burnout?

Dr: Being hungry and passionate and being happy outside of work will fix your problems at work.

**JE**

J: I first want to say thanks for taking the time to talk with me today, I really appreciate it. I want to let you know that this interview will be recorded and then transcribed. This will remain anonymous and names of people or hospitals or programs that were said will be removed. After three months, the recording will be destroyed.

J: What is your present position?

Dr: Associate director of emergency medical services.

J: How long have you been in this position?

Dr: 7 years

J: What is your age?  Gender? Ethnicity?

Dr: 42, female, chinese

J: All things considered, on a scale from 0-10, how satisfied are you with your life?” (0-10)

Dr: 9

J: All things considered, on a scale from 0-10, how satisfied are you with your career? (0-10)

Dr: 9

J: How often do you feel burned out from work?

Dr: every week

J: How often do you feel you have become more callous to people since you took this job?

Dr: every day

J: Tell me about your job? What do you like best?

Dr: I like being where people can get help and being apart of that process

J: When do you struggle the most in your career?  Can you share about that? What are some of the struggles?

Dr: Struggle through racist sexist comments because I am a female non white physician.

J: Is there anything about your job that has helped you thrive?

Dr: I have an awesome group of colleagues. I never feel alone or unsupported. My life outside of work is great so it floats me.

J: When were you the happiest in your career professionally?  Personally? Why?

Dr: Probably now for the last several years.

J: Tell me about your work-life balance? Can you share a story about a time when your work-life balance was going well?

Dr: I am very particular about my schedule. You have to make that balance very deliberate. I have done it well I think. I have kids now so it is a little trickier

J: What is your approach to money? Are you paid too much, about right, too little? Do you every worry about money? Do you have enough?

Dr: I don’t have a lot of expenses, but I make more than what my family needs. I think about right.

J: How would you describe yourself as a person? Introvert? Extrovert? Something else?

Dr: Very extroverted and very vocal, more often with my face than my words.

J: What traits do you have that helped you be successful in your job?

Dr: I am hardworking and pretty diligent and I think that saves in both medicine and administrative processes. What makes sense and what do people need is what I think about.

J: What role do friendships play in your career satisfaction?

Dr: Huge

J: What role do your family play in your career satisfaction?

Dr: Huge

J: Can you share about religion or spirituality?  I want to know about the things inside of you that helps you thrive. How was that important to you?

Dr: Perspective. Its understanding that things can always be worse and I should be thankful if it is worse for someone else.

Dr: Get the work life balance right now. You should work to live instead live to work.

J: Any other thoughts or insights that we have not covered or anything else you can think of that matters to your thriving/burnout?

**BH**

J: I first want to say thanks for taking the time to talk with me today, I really appreciate it. I want to let you know that this interview will be recorded and then transcribed. This will remain anonymous and names of people or hospitals or programs that were said will be removed. After three months, the recording will be destroyed.

J: What is your present position?

Dr: Temecula Valley ER, attending

J: How long have you been in this position?

Dr: 6 years

J: What is your age?  Gender? Ethnicity?

Dr: 39, white, male

J: All things considered, on a scale from 0-10, how satisfied are you with your life?” (0-10)

Dr: 8

J: All things considered, on a scale from 0-10, how satisfied are you with your career? (0-10)

Dr: 2

J: How often do you feel burned out from work?

Dr: every day, every shift

J: How often do you feel you have become more callous to people since you took this job?

Dr: every day

J: Tell me about your job? What do you like best?

Dr: Helping people, but it is a thankless job. No one ever thanks you

J: When do you struggle the most in your career?  Can you share about that?

Dr: Lack of patient interaction and charting. The EMRs make me struggle t

J: Is there anything about your job that has helped you thrive?

Dr: No

J: When were you the happiest in your career professionally?  Personally? Why?

Dr: Just after residency

J: Tell me about your work-life balance?

Dr: I am active. I surf, shoot guns, mountain bike.

J: What helped make your work-life balance successful?

Dr: Time off and family.

J: How would you describe yourself as a person? Introvert? Extrovert? Something else?

Dr: Extrovert, I can relate to anyone

J: What traits do you have that helped you be successful in your job?

Dr: My ability to treat everyone the same. I am pretty genuine and I don’t really hide things

J: What role do friendships play in your career satisfaction?

Dr: High, but it’s a lonely lifestyle because my friends have different

J: What role do your family play in your career satisfaction?

Dr: I do it for them, so they are the most important thing

J: Tell me about any core values that you may have?

Dr: Faith, honesty, love, humility. I treat people fairly.

J: Can you share about religion or spirituality?  I want to know about the things inside of you that helps you thrive. How was that important to you?

Dr: Having an internal perspective makes things a little easier. It just gives you a little different perspective

J: If there was a resident joining us in this conversation, what advice would you give them?

Dr: Run. Be humble and be engaged and don’t be scared to ask questions.

**VA**

J: I first want to say thanks for taking the time to talk with me today, I really appreciate it. I want to let you know that this interview will be recorded and then transcribed. This will remain anonymous and names of people or hospitals or programs that were said will be removed. After three months, the recording will be destroyed.

J: What is your present position?

Dr: Maui Memorial, Vice chairman and medical director

J: How long have you been in this position?

Dr: 5 years

J: What is your age?  Gender? Ethnicity?

Dr: thai, male, 45

J: All things considered, on a scale from 0-10, how satisfied are you with your life?” (0-10)

Dr: 7

J: All things considered, on a scale from 0-10, how satisfied are you with your career? (0-10)

Dr: 8

J: How often do you feel burned out from work?

Dr: few times a month

J: How often do you feel you have become more callous to people since you took this job?

Dr:

J: Tell me about your job?

Dr: I feel currently that I have more effect on peoples well beings and there lives.

J: When do you struggle the most in your career?  Can you share about that?

Dr: When you get your first job.

J: Is there anything about your job that has helped you thrive?

Dr: My colleagues and the administration that I am currently working with.

J: What helped the most to have a successful career?

Dr: You need to have an initial desire and this is what you want to do in your life.

J: Tell me about your work-life balance?

Dr: I have learned to keep a mindsight to prepare mentally for clinical shifts. You have to make sure that you have time off and recover and recognize when you are feeling a little burned out. You need to have down time.

J: What would you say were factors that helped you have a successful/happy work-life balance?

Dr: Fair compensation, fair/good support. You cant control it but the quality of patients.

J: What is your approach to money? Are you paid too much, about right, too little? Do you every worry about money? Do you have enough?

Dr: Somewhere between just about right and not enough depending on the day.

J: How would you describe yourself as a person? Introvert? Extrovert? Something else?

Dr: Little more introverted.

J: What traits do you have that helped you be successful in your job?

Dr: Sense of common sense and the ability to empathize and sympathize. I can put myself in his or hers position.

J: What role do friendships play in your career satisfaction?

Dr: Huge because you need people who you can trust and rely on.

J: What role do your family play in your career satisfaction?

Dr: Large role because you need a good stable home life.

J: Can you share about religion or spirituality?  I want to know about the things inside of you that helps you thrive. How was that important to you?

Dr: Religion definitely plays a large role. I feel that there is something more than what we have.

J: Can you share about hobbies or extra activities?  How important were these activities in your career satisfaction?

Dr: You need to have a hobby and need to get out.

J: If there was a resident joining us in this conversation, what advice would you give them?

Dr: There is something more important than just your career. Get out into the world and you need to have a good work life balance

J: Any other thoughts or insights that we have not covered or anything else you can think of that matters to your thriving/burnout?

**AP**

J: I first want to say thanks for taking the time to talk with me today, I really appreciate it. I want to let you know that this interview will be recorded and then transcribed. This will remain anonymous and names of people or hospitals or programs that were said will be removed. After three months, the recording will be destroyed.

J: What is your present position?

Dr: Emergency med physician at shasta

J: How long have you been in this position?

Dr: 5 years

J: What is your age?  Gender? Ethnicity?

Dr: 40, male, white

J: All things considered, on a scale from 0-10, how satisfied are you with your life?” (0-10)

Dr: 9

J: All things considered, on a scale from 0-10, how satisfied are you with your career? (0-10)

Dr: 8

J: How often do you feel burned out from work?

Dr: once a month

J: How often do you feel you have become more callous to people since you took this job?

Dr: once a month

J: Tell me about your job? What do you like best?

Dr: My coworkers

J: When do you struggle the most in your career?  Can you share about that?

Dr: When we are understaffed and there are multiple critical patients coming in at the same time

J: Is there anything about your job that has helped you thrive?

Dr: I guess the ability to take care of patients

J: What helped the most to have a successful career?

Dr: My view of a bigger picture

J: Tell me about your work-life balance?

Dr: I only work 6 days a month so I can maintain a healthy balance

J: Can you share a story about a time when your work-life balance was going well?

Dr: I am able to spend lots of time with my kinds

J: Can you share a story about a time when your work-life balance was not so great?

Dr: When I was working to many shifts

J: What is your approach to money? Are you paid too much, about right, too little? Do you every worry about money? Do you have enough?

Dr: About right. Everyone wants more money

J: How would you describe yourself as a person? Introvert? Extrovert? Something else?

Dr: In the middle leaning towards introvert.

J: What traits do you have that helped you be successful in your job?

Dr: My ability to multitask and my efficiency and a strong relationship with god.

J: What role do friendships play in your career satisfaction? Why?

Dr: The highest roles. The main thing I like about my job is hanging out with my friends at work

J: What role do your family play in your career satisfaction?

Dr: I work enough just to pay bills so I can spend time with my kids.

J: Can you share about religion or spirituality?  I want to know about the things inside of you that helps you thrive. How was that important to you?

Dr: because I have an active relationship with god, I have a sense of purpose, meaning and hope in life.

J: Can you share about hobbies or extra activities?  How important were these activities in your career satisfaction?

Dr: I mountain bike and wake surf and go to the lake several times a week. They are very important

J: If there was a resident joining us in this conversation, what advice would you give them?

Dr: Don’t buy too big of a house. Find life outside of work and remember that the ER will survive without you. Dive into a relationship with god.

**JK**

J: I first want to say thanks for taking the time to talk with me today, I really appreciate it. I want to let you know that this interview will be recorded and then transcribed. This will remain anonymous and names of people or hospitals or programs that were said will be removed. After three months, the recording will be destroyed.

J: What is your present position?

Dr: Emergency attending and medical director in corona

J: How long have you been in this position?

Dr: 1 year

J: What is your age?  Gender? Ethnicity?

Dr: 46, male, korean

J: All things considered, on a scale from 0-10, how satisfied are you with your life?” (0-10)

Dr: 10

J: All things considered, on a scale from 0-10, how satisfied are you with your career? (0-10)

Dr: 9

J: How often do you feel burned out from work?

Dr: few times a year

J: How often do you feel you have become more callous to people since you took this job?

Dr: few times a year

J: Tell me about your job? What do you like best?

Dr: Assessing people in their hour of need. I love helping people.

J: Is there anything about your job that has helped you thrive?

Dr: When you can relate to somebody in that moment of need and when you can make a connection with them it is very satisfying and fulfilling feeling. It’s the relationship you have with your patient

J: Tell me about your work-life balance?

Dr: I could probably do a better job with it but I am happy right now

J: What is your approach to money? Are you paid too much, about right, too little? Do you every worry about money? Do you have enough?

Dr: I grew up poor so when I had it in the beginning of my career, I spent it like crazy. I am comfortable right now but I can work more if I want to make more but I need to have time other than work.

J: How would you describe yourself as a person? Introvert? Extrovert? Something else?

Dr: My wife tells me I am extroverted but I think as I got older I became more introverted

J: What traits do you have that helped you be successful in your job?

Dr: The big thing is empathy and caring about other people.

J: What role do friendships play in your career satisfaction?

Dr: They are very important. Especially this year when you needed them most.

J: Can you share about religion or spirituality?  I want to know about the things inside of you that helps you thrive. How was that important to you?

Dr: I think that it helps me look at people differently.

J: If there was a resident joining us in this conversation, what advice would you give them?

Dr: Get the work life balance down.

**WY**

J: I first want to say thanks for taking the time to talk with me today, I really appreciate it. I want to let you know that this interview will be recorded and then transcribed. This will remain anonymous and names of people or hospitals or programs that were said will be removed. After three months, the recording will be destroyed.

J: What is your age?  Gender? Ethnicity?

Dr: 45, male, white

J: What is your present position?

Dr: staff emergency physician at temcual valley

J: How long have you been in this position?

Dr: 6 years

J: All things considered, on a scale from 0-10, how satisfied are you with your life?” (0-10)

Dr: 8

J: All things considered, on a scale from 0-10, how satisfied are you with your career? (0-10)

Dr: 8

J: How often do you feel burned out from work?

Dr: few times a year

J: How often do you feel you have become more callous to people since you took this job?

Dr: few times a year

J: Tell me about your job? What do you like best?

Dr: Working with people

J: When do you struggle the most in your career?  Can you share about that?

Dr: Time management

J: Is there anything about your job that has helped you thrive?

Dr: Feeling like I am making a positive difference

J: Tell me about your work-life balance?

Dr: I have more control over my schedule and chosen to work just night shifts and more time with my family when they are awake.

J: Can you share a story about a time when your work-life balance was not so great?

Dr: When I was in residency I worked more than I wanted to and no control over my schedule and like I was getting stressed and burned out

J: What is your approach to money? Are you paid too much, about right, too little? Do you every worry about money? Do you have enough?

Dr: I am lucky and blessed. I make the money I have earned. I would be doing the same job for less money so I guess I am over payed but I don’t feel guilty for the income that I make

J: How would you describe yourself as a person? Introvert? Extrovert? Something else?

Dr: I am not in either category, somewhere in the middle. You have to put yourself forward as a leader in a team and you need extrovert characteristics

J: What traits do you have that helped you be successful in your job?

Dr: Very patient and try to be a good communicator

J: What role do friendships play in your career satisfaction?

Dr: Somewhat important that you like who you work with

J: What role do your family play in your career satisfaction?

Dr: Somewhat important role that my kids think I am doing a good job. Also, raising kids has made me a much better doctor

J: Tell me about any core values that you may have?

Dr: Honor courage and commitment.

J: Can you share about religion or spirituality?  I want to know about the things inside of you that helps you thrive. How was that important to you?

Dr: I try to use religion in connecting with people and trying to influence them in making good choices. It isn’t a driving force for me.

J: Can you share about hobbies or extra activities?  How important were these activities in your career satisfaction?

Dr: Camping. It is not very important

J: If there was a resident joining us in this conversation, what advice would you give them?

Dr: Stick with it, it gets a lot better.

J: Any other thoughts or insights that we have not covered or anything else you can think of that matters to your thriving/burnout?

Dr: No

**BS**

J: I first want to say thanks for taking the time to talk with me today, I really appreciate it. I want to let you know that this interview will be recorded and then transcribed. This will remain anonymous and names of people or hospitals or programs that were said will be removed. After three months, the recording will be destroyed.

J: What is your age?  Gender? Ethnicity?

Dr: 33, male, mixed

J: What is your present position?

Dr: ER attending at sentanella

J: How long have you been in this position?

Dr: 1 month

J: All things considered, on a scale from 0-10, how satisfied are you with your life?” (0-10)

Dr: 10

J: All things considered, on a scale from 0-10, how satisfied are you with your career? (0-10)

Dr: 8

J: How often do you feel burned out from work?

Dr: once

J: How often do you feel you have become more callous to people since you took this job?

Dr: I dont

J: Tell me about your job? What do you like best?

Dr: I like the pace and how I can help people and knowledge and skill base that a lot of people don’t. I like to leave work at work when I go home.

J: When do you struggle the most in your career?  Can you share about that?

Dr: The EMR. Administrative things like if certain processes are slowing me down.

J: Is there anything about your job that has helped you thrive?

Dr: No

J: When were you the happiest in your career professionally?  Personally? Why?

Dr: Rigth now because all of my hard work is getting paid off

J: Tell me about your work-life balance?

Dr: I deal a lot with the schedulers and I like to give myself enough time off. I need to make plans for days off ahead of time and so you can look forward to it.

J: Can you share a story about a time when your work-life balance was not so great?

Dr: Very hard during all of residency

J: What is your approach to money? Are you paid too much, about right, too little? Do you every worry about money? Do you have enough?

Dr: About right for where I am right now. I do have enough money

J: How would you describe yourself as a person? Introvert? Extrovert? Something else?

Dr: Ambivert. We are in a business of service so you have to be able to communicate and work well with others so you need to be able to do that.

J: What traits do you have that helped you be successful in your job?

Dr: Charismatic and understanding of different cultures. Being mixed I can understand other people better I think

J: What role do friendships play in your career satisfaction?

Dr: I don’t know if they have much of an effect in the work environment yet

J: What role do your family play in your career satisfaction?

Dr: Very important and great to vent to and help with the work life balance

J: Can you share about religion or spirituality?  I want to know about the things inside of you that helps you thrive. How was that important to you?

Dr: I don’t practice Muslim but I maintain a level of spirituality. I like to remove that when dealing with people so I try to be a good and understanding an empathetic person.

J: Can you share about hobbies or extra activities?  How important were these activities in your career satisfaction?

Dr: I just bought a house and enjoying renovating it. I am very big on fitness. Food, beaches, hiking. Very important in my career satisfaction. I picked where I wanted to live before I wanted to work.

J: If there was a resident joining us in this conversation, what advice would you give them?

Dr: read as much as you can because you can never read enough. Do a lot of questions and ask a lot of them. When you are on your own you can’t ask questions anymore. Do as much as you can

J: Any other thoughts or insights that we have not covered or anything else you can think of that matters to your thriving/burnout?

**DrN**

J: I first want to say thanks for taking the time to talk with me today, I really appreciate it. I want to let you know that this interview will be recorded and then transcribed. This will remain anonymous and names of people or hospitals or programs that were said will be removed. After three months, the recording will be destroyed.

J: What is your age?  Gender? Ethnicity?

Dr: 37, Indian, male

J: What is your present position?

Dr: ER physician in atlanta, GA

J: How long have you been in this position?

Dr: 4 years

J: All things considered, on a scale from 0-10, how satisfied are you with your life?” (0-10)

Dr: 7

J: All things considered, on a scale from 0-10, how satisfied are you with your career? (0-10)

Dr: 7

J: How often do you feel burned out from work?

Dr: once a month

J: How often do you feel you have become more callous to people since you took this job?

Dr: once a month

J: Tell me about your job? What do you like best?

Dr: I like the procedures and the subject matter. Work stays at work.

J: Is there anything about your job that has helped you thrive?

Dr: I am very good at multi tasking in real life and little bit of OCD

J: When were you the happiest in your career professionally?  Personally? Why?

Dr: First moved to atlanta, and when I had less administrative role

J: Tell me about your work-life balance?

Dr: I try to go to the gym on my days off but I feel like I don’t have a day off. When I am not working, I have other work because of the administrative role.

J: Can you share a story about a time when your work-life balance was not so great?

Dr: It has been bad since medical school. 13 years I have had no life

J: What is your approach to money? Are you paid too much, about right, too little? Do you every worry about money? Do you have enough?

Dr: Paid very well but too little for what I do. I have plenty of money

J: How would you describe yourself as a person? Introvert? Extrovert? Something else?

Dr: extrovert

J: What traits do you have that helped you be successful in your job?

Dr: I get along with people really well. Also an intense person

J: What role do friendships play in your career satisfaction?

Dr: It is important.

J: What role do your family play in your career satisfaction?

Dr: No role

J: Tell me about any core values that you may have?

Dr: I am a very honest person and caring. Integrity and I am always a man of my word.

J: Can you share about religion or spirituality?  I want to know about the things inside of you that helps you thrive. How was that important to you?

Dr: I am very rational thinking but I am not spiritual or religious

J: Can you share about hobbies or extra activities?  How important were these activities in your career satisfaction?

Dr: Tennis, racquet ball, cross fit, gym. Extremely important

J: If there was a resident joining us in this conversation, what advice would you give them?

Dr: Pick something that you find very interesting definitely pick a career that has not been taken over by corporations

J: Any other thoughts or insights that we have not covered or anything else you can think of that matters to your thriving/burnout?

**SU**

J: I first want to say thanks for taking the time to talk with me today, I really appreciate it. I want to let you know that this interview will be recorded and then transcribed. This will remain anonymous and names of people or hospitals or programs that were said will be removed. After three months, the recording will be destroyed.

J: What is your age?  Gender? Ethnicity?

Dr: Female, Caucasian

J: What is your present position?

Dr: Attending at two different emergency rooms

J: How long have you been in this position?

Dr: 9

J: All things considered, on a scale from 0-10, how satisfied are you with your life?” (0-10)

Dr: 8

J: All things considered, on a scale from 0-10, how satisfied are you with your career? (0-10)

Dr: 9

J: How often do you feel burned out from work?

Dr: few times a year

J: How often do you feel you have become more callous to people since you took this job?

Dr: not often

J: Tell me about your job? What do you like best?

Dr: The variety in medicine

J: When do you struggle the most in your career?  Can you share about that?

Dr: When I feel like I cant practice the medicine I want to practice.

J: Is there anything about your job that has helped you thrive?

Dr: I thrive when I feel good about what I am doing which has been the most of my career. It is almost instant gratification and you know you are making an impact

J: When were you the happiest in your career professionally?  Personally? Why?

Dr: When I was a teaching attending.

J: What helped the most to have a successful career?

Dr: Balance and that is why I picked emergency medicine. I know when I need a break and I know I can get it easily.

J: Tell me about your work-life balance?

Dr: Very important. I do self-care. It is a work hard play hard mentality. It is just knowing when I need to take a break and then I just take that break.

J: Can you share a story about a time when your work-life balance was not so great?

Dr: Early in my career I was working a lot and suddenly after making no money you start making money. I was having relationship problems which was effecting my work so I took two months off to figure that out and I can do that because I am an ER doc.

J: What is your approach to money? Are you paid too much, about right, too little? Do you every worry about money? Do you have enough?

Dr: Too little. I make a lot of money but I live in the most expensive places in the country. I have enough money and I have children and worry about college but I feel okay about it.

J: How would you describe yourself as a person? Introvert? Extrovert? Something else?

Dr: Extrovert, I like being around people but I am great on my own.

J: What traits do you have that helped you be successful in your job?

Dr: I am good at multitasking

J: What role do friendships play in your career satisfaction?

Dr: Huge. Friendship for me is a life-line.

J: Tell me about any core values that you may have?

Dr: I believe in loyalty and honestly and transparency in relationship. I am a rule follower for the most part and I try to always do the right thing and be kind and inclusive as much as you can.

J: Can you share about religion or spirituality?  I want to know about the things inside of you that helps you thrive. How was that important to you?

Dr: I grew jewish but now I am much more spiritual. I am not observant but grateful to what I was taught. Family is a focus and that gives me a lot of strengths

J: Can you share about hobbies or extra activities?  How important were these activities in your career satisfaction?

Dr: I spin a lot. I cant survive without hobbies outside of work.

J: If there was a resident joining us in this conversation, what advice would you give them?

Dr: Make sure you get to know yourself as a physician and what makes you happy in your career and balance your life. You need not notice if you fall off the wagon and know you can stop and change that.

J: Any other thoughts or insights that we have not covered or anything else you can think of that matters to your thriving/burnout?

**MV**

J: I first want to say thanks for taking the time to talk with me today, I really appreciate it. I want to let you know that this interview will be recorded and then transcribed. This will remain anonymous and names of people or hospitals or programs that were said will be removed. After three months, the recording will be destroyed.

J: What is your age?  Gender? Ethnicity?

Dr: 65, male, white

J: What is your present position?

Dr: Staff ER doc at seton coast side

J: How long have you been in this position?

Dr: 18 years

J: All things considered, on a scale from 0-10, how satisfied are you with your life?” (0-10)

Dr: 7

J: All things considered, on a scale from 0-10, how satisfied are you with your career? (0-10)

Dr: 7

J: How often do you feel burned out from work?

Dr: once a month

J: How often do you feel you have become more callous to people since you took this job?

Dr: never

J: Tell me about your job? What do you like best?

Dr: Dealing with true emergency cases and treating it especially if the treatment is successful

J: When do you struggle the most in your career?  Can you share about that?

Dr: With patients, trivial or invented complaints who give virtually no possibility of successful outcome.

J: Is there anything about your job that has helped you thrive?

Dr: The flexibility of hours

J: When were you the happiest in your career professionally?  Personally? Why?

Dr: Shortly after beginning.

J: What helped the most to have a successful career?

Dr: Rarely having to aggressively look for new positions

J: Tell me about your work-life balance?

Dr: At this point, we work 24 hour shifts in a low volume facility which means at the end of that shift the compensation is pretty close to one where…

J: Can you share a story about a time when your work-life balance was not so great?

Dr: 20 years ago after working full time ER I was quite dissatisfied and ER in general and went through a two year training program and establishing a practice when I was working part time ER.

J: What is your approach to money? Are you paid too much, about right, too little? Do you every worry about money? Do you have enough?

Dr: About right. I have enough

J: How would you describe yourself as a person? Introvert? Extrovert? Something else?

Dr: Extrovert at work and introvert in personal life.

J: What traits do you have that helped you be successful in your job?

Dr: integrity and honesty and compassion

J: What role do friendships play in your career satisfaction?

Dr: moderate degree

J: What role do your family play in your career satisfaction?

Dr: The most important to me

J: Tell me about any core values that you may have?

Dr: honesty and the golden rule

J: Can you share about hobbies or extra activities?  How important were these activities in your career satisfaction?

Dr: Professionally musician. Extremely important to me, it makes working as a doc less onerous

J: If there was a resident joining us in this conversation, what advice would you give them?

Dr: Do what it expected of you and keep a low profile. If you are interested, in medicine, avoid any administrative roles.

J: Any other thoughts or insights that we have not covered or anything else you can think of that matters to your thriving/burnout?

**PS**

J: I first want to say thanks for taking the time to talk with me today, I really appreciate it. I want to let you know that this interview will be recorded and then transcribed. This will remain anonymous and names of people or hospitals or programs that were said will be removed. After three months, the recording will be destroyed.

J: What is your age?  Gender? Ethnicity?

Dr: 37, male, sri lankan

J: What is your present position?

Dr: assistant clinical professor and attending

J: How long have you been in this position?

Dr: 4

J: All things considered, on a scale from 0-10, how satisfied are you with your life?” (0-10)

Dr: 8

J: All things considered, on a scale from 0-10, how satisfied are you with your career? (0-10)

Dr: 7

J: How often do you feel burned out from work?

Dr: few times a year

J: How often do you feel you have become more callous to people since you took this job?

Dr: few times a year

J: Tell me about your job? What do you like best?

Dr: Very different patient populations. I enjoy the collegiality between the staff and everyone at the hospitals.

J: When do you struggle the most in your career?  Can you share about that?

Dr: It varies with time and there are different phases through residency. I think I am in a phase when it is more the systems issues that bother me, like the charting, and failure in the system the prevent us from providing care and resources to people.

J: Is there anything about your job that has helped you thrive?

Dr: The colleagues and comradery and the patients and see what they are going through

J: When were you the happiest in your career professionally?  Personally? Why?

Dr: I am pretty happy now. I have a lot of control over my schedule so I appreciate that. I feel good about my career right now.

J: Tell me about your work-life balance?

Dr: Having control over the schedule. So I have a decent amount of time off and spend time with my wife and do regular things. I can work as little or as much as I want

J: Can you share a story about a time when your work-life balance was not so great?

Dr: Residency because you don’t have any control over your schedule

J: What is your approach to money? Are you paid too much, about right, too little? Do you every worry about money? Do you have enough?

Dr: It varies between the places I work.

J: How would you describe yourself as a person? Introvert? Extrovert? Something else?

Dr: I am a very social person and seeing people but I am not a very loud person. I don’t need to be the center of the room but I enjoy interacting with people for sure.

J: What traits do you have that helped you be successful in your job?

Dr: I am stoic and calm. Creating environment that is calm helps a lot

J: What role do friendships play in your career satisfaction?

Dr: Pretty significant role.

J: What role do your family play in your career satisfaction?

Dr: Big as well

J: Can you share about religion or spirituality?  I want to know about the things inside of you that helps you thrive. How was that important to you?

Dr: Religion isn’t a huge role. I try to keep a perspective that there is something greater than me and I don’t have all the answers.

J: Can you share about hobbies or extra activities?  How important were these activities in your career satisfaction?

Dr: A lot of social things and interacting with other people. I don’t have as many solo hobbies. They are super important to be able to have those opportunities to do that.

J: If there was a resident joining us in this conversation, what advice would you give them?

Dr: Figure out your priorities and residency is a fixed thing, You will figure out your work life balance

**MC**

J: I first want to say thanks for taking the time to talk with me today, I really appreciate it. I want to let you know that this interview will be recorded and then transcribed. This will remain anonymous and names of people or hospitals or programs that were said will be removed. After three months, the recording will be destroyed.

J: What is your age?  Gender? Ethnicity?

Dr: 55, male, Mexican american

J: What is your present position?

Dr: working physician and ED director in Montclair CA

J: How long have you been in this position?

Dr: 25 years

J: All things considered, on a scale from 0-10, how satisfied are you with your life?” (0-10)

Dr: 9

J: All things considered, on a scale from 0-10, how satisfied are you with your career? (0-10)

Dr: 8

J: How often do you feel burned out from work?

Dr: once a month

J: How often do you feel you have become more callous to people since you took this job?

Dr: not often

J: Tell me about your job? What do you like best?

Dr: Treating patients and people and don’t like the administrative role as much

J: When do you struggle the most in your career?  Can you share about that?

Dr: Inefficiency within the ED

J: Is there anything about your job that has helped you thrive?

Dr: Managing my ED work hours and then having my family and family life.

J: When were you the happiest in your career professionally?  Personally? Why?

Dr: Now. Despite pandemic, work as managed to help micro manage our sites to where we feel we are taken care of and take care of patients safely.

J: Tell me about your work-life balance?

Dr: I can set my schedule so I don’t like to work too many days in a row. I plan to get a lot of rest. I don’t over work in terms of my hours.

J: Can you share a story about a time when your work-life balance was not so great?

Dr: No, actually.

J: What is your approach to money? Are you paid too much, about right, too little? Do you every worry about money? Do you have enough?

Dr: I think I am underpaid

J: How would you describe yourself as a person? Introvert? Extrovert? Something else?

Dr: I think I am introvert but I can be extroverted when I have to be.

J: What traits do you have that helped you be successful in your job?

Dr: I work really hard so I was raised with a strong work ethic.

J: What role do friendships play in your career satisfaction?

Dr: Significant role and you have to get along with them.

J: What role do your family play in your career satisfaction?

Dr: They support everything I do and my wife is in medicine so we always support each other.

J: Can you share about religion or spirituality?  I want to know about the things inside of you that helps you thrive. How was that important to you?

Dr: I don’t think I believe in religion at this point because I am more rooted in science and the more I see people voicing their politic

J: Can you share about hobbies or extra activities?  How important were these activities in your career satisfaction?

Dr: I like to stay active. And they are very important

J: If there was a resident joining us in this conversation, what advice would you give them?

Dr: Don’t focus on money and paying off loans right away because you will over work and then get burned out.

J: Any other thoughts or insights that we have not covered or anything else you can think of that matters to your thriving/burnout?

**BG**

J: I first want to say thanks for taking the time to talk with me today, I really appreciate it. I want to let you know that this interview will be recorded and then transcribed. This will remain anonymous and names of people or hospitals or programs that were said will be removed. After three months, the recording will be destroyed.

J: What is your age?  Gender? Ethnicity?

Dr: 34, female, white

J: What is your present position?

Dr: attending ER at corona

J: How long have you been in this position?

Dr: almost 3 years

J: All things considered, on a scale from 0-10, how satisfied are you with your life?” (0-10)

Dr: 9

J: All things considered, on a scale from 0-10, how satisfied are you with your career? (0-10)

Dr: 8

J: How often do you feel burned out from work?

Dr: few times a month

J: How often do you feel you have become more callous to people since you took this job?

Dr: not now but a couple times a month

J: Tell me about your job?

Dr: I love the people that I work with and my coworkers and doing the actual work and helping people.

J: When do you struggle the most in your career?  Can you share about that?

Dr: Some of the patients don’t have realistic expectations and sometimes those are difficult to navigate. Also sexism is a problem for me.

J: Is there anything about your job that has helped you thrive?

Dr: Just getting to talk to so many people has helped me have empathy with patients. Every case is an opportunity.

J: When were you the happiest in your career professionally?  Personally? Why?

Dr: I have only been an attending for four years but I will probably say now.

J: Tell me about your work-life balance?

Dr: Its all about marrying the right person. My husband is so supportive. I couldn’t have the family part without him. I have a lot of help that helps with the balance

J: Can you share a story about a time when your work-life balance was not so great?

Dr: After residency and moving back to home town and thinking our parents were going to help us with the kids.

J: What is your approach to money? Are you paid too much, about right, too little? Do you every worry about money? Do you have enough?

Dr: Fairly compensated

J: How would you describe yourself as a person? Introvert? Extrovert? Something else?

Dr: Pretty extroverted. I am sarcastic and joke around a lot. I love my friend group and I am giver. I love to give. Very laid back and not rigid in my thinking.

J: What traits do you have that helped you be successful in your job?

Dr: Not being an asshole. I am a very handworker. I am not afraid of failure. You need a thick skin because people will be evaluating you all the time.

J: What role do friendships play in your career satisfaction?

Dr: Very important. My best friends have come from work and I lean on them and talk to them.

J: Can you share about religion or spirituality?  I want to know about the things inside of you that helps you thrive. How was that important to you?

Dr: I don’t have as strong in my life. The science did that to me. It doesn’t play a big role but I support people who do that.

J: Can you share about hobbies or extra activities?  How important were these activities in your career satisfaction?

Dr: Go to the spa. I am a mom when I am not at work. I enjoy keeping little humans alive. Watching any trashy reality tv shows.

J: If there was a resident joining us in this conversation, what advice would you give them?

Dr: Keep your head up and work hard and keep a good attitude and don’t let the negative comments define who you are.

J: Any other thoughts or insights that we have not covered or anything else you can think of that matters to your thriving/burnout?

**JR**

J: I first want to say thanks for taking the time to talk with me today, I really appreciate it. I want to let you know that this interview will be recorded and then transcribed. This will remain anonymous and names of people or hospitals or programs that were said will be removed. After three months, the recording will be destroyed.

J: What is your age?  Gender? Ethnicity?

Dr: 44, male, white

J: What is your present position?

Dr: maui memorial, emergency physician

J: How long have you been in this position?

Dr: 2 and a half years

J: All things considered, on a scale from 0-10, how satisfied are you with your life?” (0-10)

Dr: 9

J: All things considered, on a scale from 0-10, how satisfied are you with your career? (0-10)

Dr: 7

J: How often do you feel burned out from work?

Dr: once a year

J: How often do you feel you have become more callous to people since you took this job?

Dr: much more callous

J: Tell me about your job? What do you like best?

Dr: I love not knowing what is coming through the door. I love very sick people and making them better

J: When do you struggle the most in your career?  Can you share about that?

Dr: When there is a really sick kid

J: Is there anything about your job that has helped you thrive?

Dr: ??

J: When were you the happiest in your career professionally?  Personally? Why?

Dr: Right now pre covid.

J: What helped the most to have a successful career?

Dr: I am able to boil things down to the most important parts and not get distracted by the nonsense. I am most successful here.

J: Tell me about your work-life balance?

Dr: Here I work less than a 100 hours per month and I can be with my family more.

J: Can you share a story about a time when your work-life balance was not so great?

Dr: Right out of residency I worked like 22 shifts a month and night shifts and I was exhausted and grumpy but I had to do it,

J: What is your approach to money? Are you paid too much, about right, too little? Do you every worry about money? Do you have enough?

Dr: I get paid too much.

J: How would you describe yourself as a person? Introvert? Extrovert? Something else?

Dr: Not introvert but I like my alone time. I am pretty social.

J: What role do friendships play in your career satisfaction?

Dr: Without them I would be pretty miserable.

J: What role do your family play in your career satisfaction?

Dr: Also a big role.

J: Can you share about religion or spirituality?  I want to know about the things inside of you that helps you thrive. How was that important to you?

Dr: I am not a religious person but at the same time I am a spiritual person and I believe in Karma.

J: Can you share about hobbies or extra activities?  How important were these activities in your career satisfaction?

Dr: Bunch of random stuff and they play a huge role in my career satisfaction

J: If there was a resident joining us in this conversation, what advice would you give them?

Dr: have something outside of medicine that you love because without that, you will fall apart

J: Any other thoughts or insights that we have not covered or anything else you can think of that matters to your thriving/burnout?

Dr: Have a schedule that works for you.

**GAS**

J: I first want to say thanks for taking the time to talk with me today, I really appreciate it. I want to let you know that this interview will be recorded and then transcribed. This will remain anonymous and names of people or hospitals or programs that were said will be removed. After three months, the recording will be destroyed.

J: What is your age?  Gender? Ethnicity?

Dr: 49, female, middle eastern

J: What is your present position?

Dr: er doc at two hospitals.

J: How long have you been in this position?

Dr: 22 years

J: All things considered, on a scale from 0-10, how satisfied are you with your life?” (0-10)

Dr: 9

J: All things considered, on a scale from 0-10, how satisfied are you with your career? (0-10)

Dr: 9

J: How often do you feel burned out from work?

Dr: once a year

J: How often do you feel you have become more callous to people since you took this job?

Dr: not often

J: Tell me about your job?

Dr: a privilege to be there when people are hurting. You can be there for someone and turn a really bad experience into a really good one for them. I love the connection and being there for patients and making medicine understandable . I like making people smile and saying “welcome to the body shop, I will be your mechanic”. What is there not to love?

J: Is there anything about your job that has helped you thrive?

Dr: The ability to manage what is thrown at me. Having to learn to be stable in the eye of the storm that many people cant relate to

J: Tell me about your work-life balance?

Dr: I make sure to get outside and get some sunlight. At least two or three days I go to the beach and lay in the sun to feel alive. I dance in the kitchen and I make time for friends and zoom connections. I love to read and make sure I read all kinds of things. I am a whole human being and not just a doctor.

J: How would you describe yourself as a person? Introvert? Extrovert? Something else?

Dr: I am an introverted extrovert. I was so shy when I started

J: What traits do you have that helped you be successful in your job?

Dr: being an empath because we tend to notice things more closely. Compassion and neutrality. Learning the true compassion comes from neutrality.

J: What role do friendships play in your career satisfaction?

Dr: Connection for me is highest value. Wherever I work they become my family. I like to remember personal things about them, without the human touch, life has no meaning for me. Me being with a friend is soul food.

J: Tell me about any core values that you may have?

Dr: Integrity – I do what I say and I am the same person in all situations and people know what they are getting with me. Sense of purpose bigger than money. Spirituality. Communication. Financially sound and generous.

J: Can you share about religion or spirituality?  I want to know about the things inside of you that helps you thrive. How was that important to you?

Dr: Spirituality for me is the faith in something bigger than my self and a sense of purpose and being part of a greater whole. Karma and actions have consequences helps me to feel that even if the person in front of me doesn’t reciprocate. Spirituality informs my work because it comes down to my whole motto which is “leave every life better for having been in it”. Cooking dinner for a friend or whatever

J: Can you share about hobbies or extra activities?  How important were these activities in your career satisfaction?

Dr: I love to cook and spend time with my friends and dance and read and love to travel and learn new things and always a life long learner. I tend to say I work to live – travel is very important. I work hard and play hard. All are so important to me – the oil in the engine – all critical for career satisfaction

J: If there was a resident joining us in this conversation, what advice would you give them?

Dr: Figure out why you became a doctor. Tell them to let go of everything they think they need to be in order to be one. Tell them to learn to be okay with saying I don’t know and not putting up a front. And to allow themselves to be human and learn to be present without being attached. The art of being 100% committed to a patient without feeling attached.

J: Any other thoughts or insights that we have not covered or anything else you can think of that matters to your thriving/burnout?

Dr: Yes. Find out why you became a doctor and find out the feelings you wanted to go from being a doctor. If you are feeling burnt out, figuring out why you aren’t getting your needs met.
