# Supplementary material for: Job and life satisfaction among emergency physicians: A qualitative study
Source: PLoS One. 2023 Feb 24;18(2):e0279425. doi: 10.1371/journal.pone.0279425 (PMC9955602; doi:10.1371/journal.pone.0279425)
Supplement: Attachment — Submitted filename: 2022 Mar - PLOS ONE Authors Response.docx [file pone.0279425.s003.docx]

PONE-D-22-01345

Job and Life Satisfaction Among Emergency Physicians: a Qualitative Study

PLOS ONE

Dear Dr. Doolittle,

Thank you for submitting your manuscript to PLOS ONE. After careful consideration, we feel that it has merit but does not fully meet PLOS ONE’s publication criteria as it currently stands. Therefore, we invite you to submit a revised version of the manuscript that addresses the points raised during the review process.

We see value in this manuscript. However, the referees have raised some important concerns in regard to experimental design, presentation of the paper contribution and literature review. Thus, we ask that you carefully revise your manuscript along the lines they have suggested.
In addition, it will be important that you explicitly acknowledge the limitations and assumptions underlying your method and conclusions.

Please submit your revised manuscript by Apr 10 2022 11:59PM. If you will need more time than this to complete your revisions, please reply to this message or contact the journal office at [plosone@plos.org](mailto:plosone@plos.org). When you're ready to submit your revision, log on to https://www.editorialmanager.com/pone/ and select the 'Submissions Needing Revision' folder to locate your manuscript file.

Please include the following items when submitting your revised manuscript:

- A rebuttal letter that responds to each point raised by the academic editor and reviewer(s). You should upload this letter as a separate file labeled 'Response to Reviewers'.
- A marked-up copy of your manuscript that highlights changes made to the original version. You should upload this as a separate file labeled 'Revised Manuscript with Track Changes'.
- An unmarked version of your revised paper without tracked changes. You should upload this as a separate file labeled 'Manuscript'.

If you would like to make changes to your financial disclosure, please include your updated statement in your cover letter. Guidelines for resubmitting your figure files are available below the reviewer comments at the end of this letter.

If applicable, we recommend that you deposit your laboratory protocols in protocols.io to enhance the reproducibility of your results. Protocols.io assigns your protocol its own identifier (DOI) so that it can be cited independently in the future. For instructions see: [https://journals.plos.org/plosone/s/submission-guidelines#loc-laboratory-protocols](https://nam12.safelinks.protection.outlook.com/?url=https%3A%2F%2Fjournals.plos.org%2Fplosone%2Fs%2Fsubmission-guidelines%23loc-laboratory-protocols&data=04%7C01%7Cbenjamin.doolittle%40yale.edu%7C507ed29ead2447ec35e908d9f7b0cbf5%7Cdd8cbebb21394df8b4114e3e87abeb5c%7C0%7C0%7C637813162216609879%7CUnknown%7CTWFpbGZsb3d8eyJWIjoiMC4wLjAwMDAiLCJQIjoiV2luMzIiLCJBTiI6Ik1haWwiLCJXVCI6Mn0%3D%7C3000&sdata=nwhXFfYijrpMAgf47KRnTYQNw8ATkAS4%2FpNBNOvQyiY%3D&reserved=0). Additionally, PLOS ONE offers an option for publishing peer-reviewed Lab Protocol articles, which describe protocols hosted on protocols.io. Read more information on sharing protocols at [https://plos.org/protocols?utm_medium=editorial-email&utm_source=authorletters&utm_campaign=protocols](https://nam12.safelinks.protection.outlook.com/?url=https%3A%2F%2Fplos.org%2Fprotocols%3Futm_medium%3Deditorial-email%26utm_source%3Dauthorletters%26utm_campaign%3Dprotocols&data=04%7C01%7Cbenjamin.doolittle%40yale.edu%7C507ed29ead2447ec35e908d9f7b0cbf5%7Cdd8cbebb21394df8b4114e3e87abeb5c%7C0%7C0%7C637813162216609879%7CUnknown%7CTWFpbGZsb3d8eyJWIjoiMC4wLjAwMDAiLCJQIjoiV2luMzIiLCJBTiI6Ik1haWwiLCJXVCI6Mn0%3D%7C3000&sdata=%2BhgLDH28pZVLS9DxAXNeUCKtatMaRXcyzraHl3F4ylc%3D&reserved=0).

We look forward to receiving your revised manuscript.

Kind regards,

Andrea Fronzetti Colladon, Ph.D.
Academic Editor
PLOS ONE

**AUTHOR RESPONSE**

Journal Requirements:

When submitting your revision, we need you to address these additional requirements.

1. Please ensure that your manuscript meets PLOS ONE's style requirements, including those for file naming. The PLOS ONE style templates can be found at
   <https://journals.plos.org/plosone/s/file?id=wjVg/PLOSOne_formatting_sample_main_body.pdf>
2. <https://journals.plos.org/plosone/s/file?id=ba62/PLOSOne_formatting_sample_title_authors_affiliations.pdf>.

2. Please provide additional details regarding participant consent. In the ethics statement in the Methods and online submission information, please ensure that you have specified (1) whether consent was informed and (2) what type you obtained (for instance, written or verbal, and if verbal, how it was documented and witnessed). If your study included minors, state whether you obtained consent from parents or guardians. If the need for consent was waived by the ethics committee, please include this information.

If you are reporting a retrospective study of medical records or archived samples, please ensure that you have discussed whether all data were fully anonymized before you accessed them and/or whether the IRB or ethics committee waived the requirement for informed consent. If patients provided informed written consent to have data from their medical records used in research, please include this information.

*We obtained both verbal and written consent which detail in our Methods section. In addition, we note, “The interviews were recorded and transcribed. Any identifying data was removed from the transcription to preserve anonymity.” In addition, we add the number of the Yale University Institutional Review Board approved protocol, IRB # 2000022828.*

3. Thank you for stating the following in your Competing Interests section:

[No authors have competing interests.]
 
Please complete your Competing Interests on the online submission form to state any Competing Interests. If you have no competing interests, please state "The authors have declared that no competing interests exist.", as detailed online in our guide for authors at http://journals.plos.org/plosone/s/submit-now 
 This information should be included in your cover letter; we will change the online submission form on your behalf.

*Done. We have included the line in our cover letter, “The authors have declared that no competing interests exist.”*

4. In your Data Availability statement, you have not specified where the minimal data set underlying the results described in your manuscript can be found. PLOS defines a study's minimal data set as the underlying data used to reach the conclusions drawn in the manuscript and any additional data required to replicate the reported study findings in their entirety. All PLOS journals require that the minimal data set be made fully available. For more information about our data policy, please see http://journals.plos.org/plosone/s/data-availability.

Upon re-submitting your revised manuscript, please upload your study’s minimal underlying data set as either Supporting Information files or to a stable, public repository and include the relevant URLs, DOIs, or accession numbers within your revised cover letter. For a list of acceptable repositories, please see http://journals.plos.org/plosone/s/data-availability#loc-recommended-repositories. Any potentially identifying patient information must be fully anonymized.

Important: If there are ethical or legal restrictions to sharing your data publicly, please explain these restrictions in detail. Please see our guidelines for more information on what we consider unacceptable restrictions to publicly sharing data: http://journals.plos.org/plosone/s/data-availability#loc-unacceptable-data-access-restrictions. Note that it is not acceptable for the authors to be the sole named individuals responsible for ensuring data access.

We will update your Data Availability statement to reflect the information you provide in your cover letter.

*We have uploaded the transcripts in as a “Supporting Information” file, and note this in our cover letter.*

5. Please amend either the abstract on the online submission form (via Edit Submission) or the abstract in the manuscript so that they are identical.

*We have double checked that this is the case.*

6. Please include your full ethics statement in the ‘Methods’ section of your manuscript file. In your statement, please include the full name of the IRB or ethics committee who approved or waived your study, as well as whether or not you obtained informed written or verbal consent. If consent was waived for your study, please include this information in your statement as well.

*We added the line, “Both recorded verbal and written consent was obtained at the beginning of the interview.” In addition, we add the number of the Yale University Institutional Review Board approved protocol, IRB # 2000022828.*

Additional Editor Comments:

We see value in this manuscript. However, the referees have raised some important concerns in regard to experimental design, presentation of the paper contribution and literature review. Thus, we ask that you carefully revise your manuscript along the lines they have suggested.
In addition, it will be important that you explicitly acknowledge the limitations and assumptions underlying your method and conclusions.


[Note: HTML markup is below. Please do not edit.]

Reviewers' comments:

Reviewer's Responses to Questions

**Comments to the Author**

1. Is the manuscript technically sound, and do the data support the conclusions?

The manuscript must describe a technically sound piece of scientific research with data that supports the conclusions. Experiments must have been conducted rigorously, with appropriate controls, replication, and sample sizes. The conclusions must be drawn appropriately based on the data presented.

Reviewer #1: Yes

Reviewer #2: Yes

2. Has the statistical analysis been performed appropriately and rigorously?

Reviewer #1: N/A

Reviewer #2: Yes

3. Have the authors made all data underlying the findings in their manuscript fully available?

The [PLOS Data policy](https://nam12.safelinks.protection.outlook.com/?url=http%3A%2F%2Fwww.plosone.org%2Fstatic%2Fpolicies.action%23sharing&data=04%7C01%7Cbenjamin.doolittle%40yale.edu%7C507ed29ead2447ec35e908d9f7b0cbf5%7Cdd8cbebb21394df8b4114e3e87abeb5c%7C0%7C0%7C637813162216609879%7CUnknown%7CTWFpbGZsb3d8eyJWIjoiMC4wLjAwMDAiLCJQIjoiV2luMzIiLCJBTiI6Ik1haWwiLCJXVCI6Mn0%3D%7C3000&sdata=oM0%2BPOkET2JFOkLI2G48h%2BhrsVqaG1tibc6a3vQmuH4%3D&reserved=0) requires authors to make all data underlying the findings described in their manuscript fully available without restriction, with rare exception (please refer to the Data Availability Statement in the manuscript PDF file). The data should be provided as part of the manuscript or its supporting information, or deposited to a public repository. For example, in addition to summary statistics, the data points behind means, medians and variance measures should be available. If there are restrictions on publicly sharing data—e.g. participant privacy or use of data from a third party—those must be specified.

Reviewer #1: Yes

Reviewer #2: No

4. Is the manuscript presented in an intelligible fashion and written in standard English?

PLOS ONE does not copyedit accepted manuscripts, so the language in submitted articles must be clear, correct, and unambiguous. Any typographical or grammatical errors should be corrected at revision, so please note any specific errors here.

Reviewer #1: Yes

Reviewer #2: Yes

5. Review Comments to the Author

Please use the space provided to explain your answers to the questions above. You may also include additional comments for the author, including concerns about dual publication, research ethics, or publication ethics. (Please upload your review as an attachment if it exceeds 20,000 characters)

Reviewer #1: This is a qualitative paper regarding career and personal satisfaction in Emergency Physicians; interestingly none of the authors are emergency physicians nor work in an emergency department.

*To both reviewers, thank you for your overall support of our manuscript and for your thoughtful comments. We recognize that these are busy times. Given the level of detailed, insightful comments, you certainly spent significant time with the manuscript. Please know how deeply we appreciate your efforts. We have incorporated your suggestions throughout the manuscript and respond in detail to your comments below.*

*In particular, the other papers that you propose are excellent. Initially, we struggled with how much detail to include. We have incorporated those papers throughout the manuscript. With your suggestions, the manuscript is much stronger. Many thanks.*

*Of note, the senior author worked for 16 years – the night shift – in a community hospital emergency department. In part, this project evolved from a senior leader at the private emergency department group approaching me to explore issues of burnout and well-being.*

Abstract comments:
When was this study conducted? Given the impact of COVID on frontline healthcare workers (emergency physicians) this is critical information and the reader needs it for context.
23 physicians with male and Asian/South Asian skew in the population
States a model is proposed, however, there is no clear model presented in the paper
The conclusion in the abstract is a stretch. The data collection was not robust enough to support encouraging these qualities while fostering supportive leadership and optimizing the EMR may improve satisfaction among physicians experiencing burnout. As physicians in this cohort were not studied, it is difficult to make this conclusion.

*We have added the dates when the interviews took place (June-September 2020) and are mindful that the project took place at varying stages of the pandemic. Although, at the hospitals we studied, the COVID-19 pandemic was in a temporary lull.*

*The demographics in our sample were similar to a large 2020 data set of Emergency Physicians:* [*https://reader.elsevier.com/reader/sd/pii/S0196064420305011?token=84857CF28F19E35C6A5089F05C41DA0F98ADB6FDB30FC5974B8008292FFE9BF4C173E56DE36B4C0D8A581B431CE2344D&originRegion=us-east-1&originCreation=20220331201623*](https://reader.elsevier.com/reader/sd/pii/S0196064420305011?token=84857CF28F19E35C6A5089F05C41DA0F98ADB6FDB30FC5974B8008292FFE9BF4C173E56DE36B4C0D8A581B431CE2344D&originRegion=us-east-1&originCreation=20220331201623)

*This study showed a median age of 50, with 72% male. While ethnicity data was not included in that large study of ED physicians, national data for all physicians show 56.2% Caucasian, 17.1% Asian, 5.8% Hispanic, 5.0% Black.* [*https://www.aamc.org/data-reports/workforce/interactive-data/figure-18-percentage-all-active-physicians-race/ethnicity-2018*](https://www.aamc.org/data-reports/workforce/interactive-data/figure-18-percentage-all-active-physicians-race/ethnicity-2018)*. Another large study published in the literature (referenced by reviewer #2,* [*https://doi.org/10.1002/emp2.12546*](https://doi.org/10.1002/emp2.12546)*) 76% male, average age of 50.82, and 84% Caucasian.*

*We believe that our representation, in general, represents that demographics of the physician workforce. We have referenced these papers in our results section.*

*A cohort of 23 participants for a qualitative review is sufficient, especially when thematic saturation was reached after 10 participants. Many qualitative studies published in PLOSONE had a similar number of participants. Twenty one primary care doctors were studied in a suicide prevention project (*[*https://doi.org/10.1371/journal.pone.0242540*](https://doi.org/10.1371/journal.pone.0242540)*), and seventeen were studied in a project about supportive roles of Black and Latinx health care workers during the COVID-19 pandemic (*[*https://doi.org/10.1371/journal.pone.0262606*](https://doi.org/10.1371/journal.pone.0262606)*).*

*Since qualitative studies are designed to draw out naturally occurring themes, our recommendations are based on this input. EMR and leadership issues have been cited in other projects, which we now include in the discussion at the recommendation of reviewer #2 (*[*https://doi.org/10.1016/j.mayocp.2016.10.004*](https://doi.org/10.1016/j.mayocp.2016.10.004)*). This qualitative study affirms the importance of optimizing the EMR, workflow, and leadership.*

*Since one of the identified stressors was the lack of support and challenges around leadership and administration, we believe it is relevant to comment about this in the conclusion. We have incorporated the paper suggested by reviewer #2 and included this in the conclusion, “Shanafelt et al describes seven drivers that leaders need to address to improve physician engagement: resources, workload, control/flexibility, work-life integration, social support, meaning in work, organizational culture. This model affirms the findings derived from the lived-experience of emergency physicians in this qualitative study. In particular, optimizing the work environment with appropriate resources and workload as well as an emphasis work-place culture through responsive leadership and social support were key drivers in our model.”*

Introduction:
There are new references on burnout in emergency physicians. Consideration should be made to review some of this work and update the introduction as needed. Additionally, there are some factors in the ED that are relatively unique and impact burnout. These should be explored and discussed.
-Stehman CR, Testo Z, Gershaw RS, Kellogg AR. Burnout, Drop Out, Suicide: Physician Loss in Emergency Medicine, Part I. West J Emerg Med. 2019 May;20(3):485-494. doi: 10.5811/westjem.2019.4.40970. Epub 2019 Apr 23. Erratum in: West J Emerg Med. 2019 Aug 21;20(5):840-841. PMID: 31123550; PMCID: PMC6526882.
-West CP, Dyrbye LN, Shanafelt TD. Physician burnout: contributors, consequences and solutions. J Intern Med. 2018 Jun;283(6):516-529. doi: 10.1111/joim.12752. Epub 2018 Mar 24. PMID: 29505159.
-Ilić IM, Arandjelović MŽ, Jovanović JM, Nešić MM. Relationships of work-related psychosocial risks, stress, individual factors and burnout - Questionnaire survey among emergency physicians and nurses. Med Pr. 2017 Mar 24;68(2):167-178. English. doi: 10.13075/mp.5893.00516. Epub 2017 Mar 13. PMID: 28345677.
-Stehman CR, Clark RL, Purpura A, Kellogg AR. Wellness: Combating Burnout and Its Consequences in Emergency Medicine. West J Emerg Med. 2020 Apr 13;21(3):555-565. doi: 10.5811/westjem.2020.1.40971. PMID: 32421501; PMCID: PMC7234720.
-Watson AG, McCoy JV, Mathew J, Gundersen DA, Eisenstein RM. Impact of physician workload on burnout in the emergency department. Psychol Health Med. 2019 Apr;24(4):414-428. doi: 10.1080/13548506.2018.1539236. Epub 2018 Oct 29. PMID: 30372132.
-Truchot J, Chauvin A, Hutin A, Leredu T, Plaisance P, Yordanov Y. Burnout and satisfaction among young emergency physicians. Eur J Emerg Med. 2018 Dec;25(6):445-446. doi: 10.1097/MEJ.0000000000000526. PMID: 30379715.

*Thank you. We have expanded the introduction to include several of these citations. We thought that the West, Ilić, and first Stehman articles were the most relevant for the introduction, and we used the Truchot article to support the conclusion.*

Methods:
Study Design
Privately held physician management company - is this a CMG? Fee for service? Caps on number of patients per shift? Across the US? Where? Which regions? Urban vs suburban vs academic? 23 were interviewed out of how many possible? Can a response rate be calculated?

*We have added the line, “… contract management group that staffs several community-based hospitals in the western United States.” We felt it necessary to protect the identity of the CMG.*

What was the rationale in excluding those with burnout? Burnout can be transient and is essentially the status quo in EM. Can EM physicians not have both burnout and high career and life satisfaction? Prior work by Shanafelt about work life satisfaction and burnout would indicate this. It follows with the "work hard, play hard" model in EM. The nature of the work may cause burnout, however, the amount of off time/free time, may not mitigate the burnout but improve career/life satisfaction.

*The rationale for excluding those with burnout was to explore a model of thriving. Burnout – and its associated factors – has been well described in the literature. However, emergency physicians who are thriving – the positive deviants – are not well described. Who is happy in their life and their career? There are very few projects among physicians which explore this concept. While we recognize that physicians may be burned out at work and have high life-satisfaction, we chose to explore a model of holistic well-being, since this is not well-studied in the literature. We have clarified this in the Introduction with the line, “Given that burnout is well-described in the literature, this project explored a model of physician thriving, as in, those who are not experiencing burnout and are satisfied both in their work and their life.”*

*Your point about the “work hard, play hard” spirit of EM is a good one. In the Discussion section, we explore this idea, “While it is conceptually possible for an emergency physician to experience burnout at work and high satisfaction at home, or vice-versa, this project explored a holistic model of both high job and life satisfaction. The Institute of Healthcare Improvement supplements their Triple Aim of healthcare, quality, efficiency, and patient experience, with a fourth aim, physician work satisfaction. We chose to explore a model of high job and life satisfaction. Our model describes the importance of both work and life satisfaction. Several quantitative projects affirm the importance of a balanced work and home life among physicians.*

*Of note, we believe that the mixed-methods nature of this study is one of its strengths. Many questionnaire studies – even those by Shanafelt et al – are plagued by low response rate. Many qualitative studies do not discriminate among those who are burned out or not.*

Semi-structured interview: Who did the interviewing? What were the questions? Can you provide the interview template? Who developed the questions? Were they piloted?
Data Collection: When was this study conducted?
Demographics - what about number of children/elderly dependents in the home? The gendered expectations of women in US society greatly impacts work life balance and career satisfaction. The skew of male participants and the lack of insight into these issues negatively impacts this work. Were there any questions regarding shift number or clinical schedule? It is possible that the same individual would give different answers after 5 days off vs 5 days of working shifts.

*The first author conducted the interviews, which we now note in the Methods section. The quantitative measures (burnout and life satisfaction) are validated instruments which we cite in the Methods section. The question template was derived from the literature on well-being and burnout, the authors’ own conceptual framework, and focused on broad themes of work culture, social support, emotional coping, and other strategies. The questions are shared in the Appendix. The same template has been used in a study among primary care physicians, which is cited (#10). The open-ended nature of the questions allowed for broad responses. Of note, participants did not voluntarily share the benefits of working 5 days on and 5 days off, nor any other specific schedule arrangement.*

Results:
Burnout can be transient and wax and wane, can the same be said for thriving? Can you have burnout and be thriving? Expanded horizons highlights my question above - can one be burned out and thrive? Many considered ... what percentage? I recognize that this is a qual study but a number would be helpful.

*We recognize that we are exploring new territory here, and much is unknown. You raise a very provocative question about being burned out and still thriving, since these are slightly different constructs. As you likely know, Shanafelt’s work shows that emergency physicians are among the most burned out AND the most satisfied with work-life balance (*[*https://doi.org/10.1016/j.mayocp.2015.08.023*](https://doi.org/10.1016/j.mayocp.2015.08.023)*).*

*In an attempt to have a well-defined model, we chose to focus on those physicians who were satisfied in both career and life. Since we used snow-ball sampling where satisfied physicians referred other satisfied physicians, we do not have aggregate scores of burnout, work and life satisfaction among all the emergency physicians in the CMG.*

Social connections: again please clarify many
Resilience: this is complicated. Most physicians by definition are high resilience. There are lot of challenges in schooling, standardized testing and training and those who make it through are often categorized as high resilience. Having high resilience doesn't mean that you do not have burnout. Additionally, many psychologists and social scientists would argue that resilience is determined in early childhood and that while it may be slightly modifiable, the majority of an individual's resilience has been determined by age 5-6. Please elaborate on this. Were participants specfically questioned about resilience? Would this have created bias?
Personality traits - so what? The introduction has not set up why this would be explored nor why it matters.
Balanced perspective: were participants specifically asked about emotional coping, spirituality and religion? Why are these lumped under balances perspective? Please clarify.
Above EMR as a threat, the incorrect role (roll) is used in the text.

*You raise several points here that reflect the nature of qualitative studies from semi-structured interviews.*

*We are not testing hypotheses, such as whether resilience correlates with burnout. Instead, we are drawing conclusions based on the naturally occurring themes among physicians who are not burned out and identify with high work and life satisfaction. This leads to findings that make sense, such as the importance of resilience. Other findings may be surprising, such as the perspective of expanded horizons or spirituality, which are not often explored in large quantitative studies of physician burnout.*

*In the semi-structured interview guide, several open-ended questions are asked to elicit coping strategies such as, “What traits do you have that help you be successful in your job?”, “Is there anything about your job that has helped you thrive?” “What helps the most to have a successful career?” The art of qualitative studies is not to ask specific leading questions – about resilience for example – but rather to follow up and expand on those themes when the participant offers them.*

*Thank you for picking up the typo. The correct “role” (not roll) is used.*

Discussion:
Thriving physicians tended to have rich ... balance, perspective and resilience. This would have been far more novel and interesting if the same questions were asked of those with burnout. There has been a shift in the medical literature identifying burnout as a system issue not an individual issue but this model is heavily focused on individual characteristics. Since those with burnout have been excluded, one cannot say whether or not those physicians also have balance, perspective and resilience.
Several intervention studies .... These studies show improvement in well-being and reduced burnout. Is improved well-being the same as thriving?
The paragraph on personality type feels out of place and lacks the so what or why is this important factor.
Leadership: at what level were participants asked about? ED director? CMO? CNO? Dept Chair? Corporate group leadership? There could be high variance here depending on what level of leadership is being assessed.
"transactional leadership" seems to define the leadership style of all CMGs and hospitals to meet CMS metrics. Please elaborate on the impact of higher level leadership and transactional leadership.
Practice setting of these physicians is critical in understanding the support from professional societies/CME statement.

*There are several important questions here that we share with the reviewer. Since the purpose of a qualitative study is to explore novel themes derived from participants’ lived-experience, by definition, the granular quality of these issues may not be well-elucidated. Quantitative, survey-type, studies are also limited in this way. We believe that the strength of our mixed-methods strategy is that thriving physicians describe both intrinsic and extrinsic factors that influence their job and life satisfaction. This takes a holistic view of the emergency physician, which we believe is an important contribution that complements the work of Shanafelt, Lall, and others. The level of leadership – ED director, CMO, etc. – may vary among institution and participant perception. The signal from our participants is that leadership matters. Thus, we felt it important to include data about transactional leadership. Certainly, further study about leadership, ED setting, personality traits, team dynamics, etc. is merited.*

Is there a typo or missing word in the sentence .. Religious involvement and spirituality .... the sentence counters itself the way it is currently written.

*We have clarified the point about religious involvement to read, “Of note, our participants did not cite organized religious involvement and other formal social clubs as important to well-being, which has been noted in broader population based studies.”*

Limitations
The diversity is lacking particularly around gender, URiM status and practice location. Please expand upon these limitations.

*The diversity of our sample size generally reflects the diversity of emergency physicians, as noted in several studies that I cite above. We now note this in the limitations section with the line, “While most physicians were male and Caucasian, our sample reflects similar demographics of larger, quantitative studies.”*

Please confirm this is the first study that examines physicians who identify as highly satisfied ...

This model is not novel in medicine. Please see models from Mayo and Stanford; ACEP wellness wheel; etc. How is this model different/improved?
What is the very low response rate? <1%? How low is very low?
Lack of generalizability to other practice settings (particularly academic medicine)
Conclusions:
Are all well physicians satisfied with their career and personal lives? Can you have burnout and high job satisfaction? please elaborate. How do you cultivate these individual factors? What are your suggested strategies?

*While we believe this is the first study of this type among emergency physicians, we soften this claim by the line in the Discussion, “…we believe this is the first study that examines physicians who identify as highly satisfied both in their professional and personal lives in a qualitative study.”*

*The models derived by Mayo, Lall, Shanafelt and others are derived from large survey-based studies. Our study, to our knowledge, is the first among emergency physicians to employ qualitative studies to explore physicians who are satisfied both in work and life. The novelty is our mixed-methods approach: validated instruments combined with open-ended interviews. This allowed for some interesting findings. In particular, physicians described themselves as both introverted and extroverted. This is a difficult construct to capture in a validated survey, but precisely the sort of nuanced finding that can be elicited from interviews.*

*Since both reviewers note that it is conceptually possible to be both burned out at work, but have high life satisfaction, and vice-versa, we include in the limitations section, “Of note, this project was designed to explore a model of thriving through the lived-experience of front-line emergency physicians. It was not designed to explore factors related to burnout.”*

*We also note in our limitations section that these physicians served several community-based hospitals in the western United States, but worked for the same CMG. We have further added in this revision, “these findings may be not be generalizable to all emergency physicians.”*

Tables/figures
Consider a diagram of your proposed model

Table 1:
What about demographic characteristics of those who were excluded?
Satisfaction scale - is this a likert score?

*Yes, the satisfaction scale is a validated Likert scale, widely used in the literature. We cite this in the Methods section. Since recruitment was self-referential and through the snowball effect, only two physicians met criteria for burnout and were not included in the analysis, since this project was to build a model of physician thriving.*

Were there any individuals with disparate scores, ie high satisfied with life but moderate satisfied with career?

*No, as physicians were recruited through self-reference and snow-ball sampling, only physicians who met criteria for high life and work satisfaction, and were not experiencing burnout were included. This was to derive a model for thriving.*

Reviewer #2: This is a very interesting topic and so relevant in the time of the coronavirus pandemic. The premise of identifying characteristics of EP’s that contribute to a satisfied and thriving career is refreshing after all the negative articles surrounding EP burnout. The concept of this study is truly forward-thinking.

*Thank you!*

Your authors state that their article is among few studies focusing on satisfaction in EM, yet they did not reference a study by Lall, et al “Are emergency physicians satisfied? An analysis of operational/organization factors,” (http://doi.org/10.1002/emp2.12546) which would add to their premise that organizational factors have the largest impact on an emergency physician’s wellbeing. Also suggest adding information from the National Academy of Medicine’s conceptual model of wellness and resilience which demonstrates that although self care is important, it is the organization that determines the bulk of an individual EP’s wellness.
The sample size in this study was small , skewed toward white males, and did not include any African American participants.
In today’s political climate, I wonder if this is a diverse enough study to be relevant for publication. The conclusions drawn from the 23 participants in this study are noteworthy, but the lack of diversity of participants is problematic.

*The Lall article is excellent, and affirms our findings. Although, we recognize that Lall takes an institutional approach, based on a survey, rather than exploring personality traits and work environment in qualitative study. We believe both models complement each other. We incorporate this paper into our discussion, “In addition, Lall et al derived a model of job satisfaction from a survey-based study that identified the importance of reducing burnout, opportunities for non-clinical opportunities, optimizing work-conditions, family time, and the electronic health record, and reducing discrimination. Our qualitative study adds validity to the Shanafelt and Lall models. In addition, our study explores intrinsic features such as resilience, balanced perspective, and being both introverted and extroverted.”*

*Reviewer #1 raised the same issue about diversity and study sample. I include my response here as well:*

*The demographics in our sample were similar to a large 2020 data set of Emergency Physicians:* [*https://reader.elsevier.com/reader/sd/pii/S0196064420305011?token=84857CF28F19E35C6A5089F05C41DA0F98ADB6FDB30FC5974B8008292FFE9BF4C173E56DE36B4C0D8A581B431CE2344D&originRegion=us-east-1&originCreation=20220331201623*](https://reader.elsevier.com/reader/sd/pii/S0196064420305011?token=84857CF28F19E35C6A5089F05C41DA0F98ADB6FDB30FC5974B8008292FFE9BF4C173E56DE36B4C0D8A581B431CE2344D&originRegion=us-east-1&originCreation=20220331201623)

*This study showed a median age of 50, with 72% male. While ethnicity data was not included in that large study of ED physicians, national data for all physicians show 56.2% Caucasian, 17.1% Asian, 5.8% Hispanic, 5.0% Black.* [*https://www.aamc.org/data-reports/workforce/interactive-data/figure-18-percentage-all-active-physicians-race/ethnicity-2018*](https://www.aamc.org/data-reports/workforce/interactive-data/figure-18-percentage-all-active-physicians-race/ethnicity-2018)*. Another large study published in the literature (referenced by reviewer #2,* [*https://doi.org/10.1002/emp2.12546*](https://doi.org/10.1002/emp2.12546)*) 76% male, average age of 50.82, and 84% Caucasian.*

*We believe that our representation, in general, represents that demographics of the physician workforce. We have referenced these papers in our results section.*

*A cohort of 23 participants for a qualitative review is sufficient, especially when thematic saturation was reached after 10 participants. Many qualitative studies published in PLOSONE had a similar number of participants. Twenty one primary care doctors were studied in a suicide prevention project (*[*https://doi.org/10.1371/journal.pone.0242540*](https://doi.org/10.1371/journal.pone.0242540)*), and seventeen were studied in a project about supportive roles of Black and Latinx health care workers during the COVID-19 pandemic (*[*https://doi.org/10.1371/journal.pone.0262606*](https://doi.org/10.1371/journal.pone.0262606)*).*

*Since this was a study to explore thriving only, and not burnout, and recruitment was self-selected and via snowball sampling, only two physicians were screened, met criteria for burnout, and thus were not included in the final analysis.*

The statistical analysis performed is satisfactory although other items to include in the one table might be: age of participants, number of years practicing emergency medicine, practice setting, etc.

The manuscript is clearly written and the themes highlighted correspond to the current literature. The statement, “Data saturation was reached after the tenth interview,” seems to be a premature conclusion. The paper lists only five common themes associated with thriving EP’s. Shanafelt listed 7 drivers of engagement in physicians so I wonder why there was a discrepancy.

*As we reviewed the transcripts in an iterative fashion, the output of themes was stable after the tenth interview. From ten to twenty-three, no new themes emerged, hence the statement about data saturation.*

*Shanafelt’s paper (*[*https://doi.org/10.1016/j.mayocp.2016.10.004*](https://doi.org/10.1016/j.mayocp.2016.10.004)*) describes nine drivers of engagement – resources, workload, control/flexibility, work-life integration, social support, meaning in work, organizational culture – that pertain to institutional interventions. This model is derived from his long-standing leadership in the field and his extensive surveys. Our model is more limited in scope and is derived from the lived experienced of front-line emergency physicians. Although different, his model does resonate with our five themes of expanded horizons, social connections, resilience, balanced perspective, qualities of both introversion and extroversion.*

*To incorporate this important paper, we have included the following in the discussion, “This model affirms the findings by Shanafelt et al who describes nine drivers that leaders need to address to improve physician engagement: resources, workload, control/flexibility, work-life integration, social support, meaning in work, organizational culture. This model affirms the findings derived from the lived-experience of emergency physicians in this qualitative study. In particular, optimizing the work environment with appropriate resources and workload as well as an emphasis work-place culture through responsive leadership and social support were key drivers in our model.”*

This study would have significant impact if the number of physicians interviewed was much higher, with a diverse group so that more themes could be identified.
Since the themes are important to physicians, the manuscript could highlight them with the caveat that this is a study of 23 non-diverse physicians so may not be applicable to all physicians.

*As we note above, 23 physicians is an appropriate number for a qualitative study, especially when thematic saturation was reached after 10 participants. Several qualitative studies exploring well-being and burnout among health care providers had similar numbers. A study among emergency physician residents employed four focus groups, and did not include the total number of participants (*[*https://doi.org/10.1080/10401334.2021.1875833*](https://doi.org/10.1080/10401334.2021.1875833)*); a study among physicians in rural Canada had 14 participants (*[*https://doi.org/10.1186/s12913-021-06899-y*](https://doi.org/10.1186/s12913-021-06899-y)*); a study among primary care physicians’ satisfaction with telehealth during the COVID-19 pandemic had 15 participants.*

*The question of diversity is valid. However, as noted above, most emergency physicians are male. The ethnic diversity of our cohort is similar to the overall diversity of physicians in the United States. We note three papers (see above) where the diversity of our sample was similar to other published studies. We now cite these in the Results and Discussion section. We also note in the Limitations section, “While most physicians were male and Caucasian, our sample reflects similar demographics of larger, quantitative studies.”*

*Many thanks for your thoughtful review and support of this project. You both raised excellent points. We hope that we have responded thoughtfully and completely to each of them. We have incorporated nearly all the articles you cite into the manuscript, softened some of our claims, and expanded both the introduction and discussion. All the best….*
