# Supplementary material for: Job and life satisfaction among emergency physicians: A qualitative study
Source: PLoS One. 2023 Feb 24;18(2):e0279425. doi: 10.1371/journal.pone.0279425 (PMC9955602; doi:10.1371/journal.pone.0279425)
Supplement: Attachment — Submitted filename: 2022 June 11 - Authors response to reviewers.docx [file pone.0279425.s004.docx]

**RESPONSE TO REVIEWERS**

Reviewers' comments:

Reviewer's Responses to Questions

Comments to the Author

1. If the authors have adequately addressed your comments raised in a previous round of review and you feel that this manuscript is now acceptable for publication, you may indicate that here to bypass the “Comments to the Author” section, enter your conflict of interest statement in the “Confidential to Editor” section, and submit your "Accept" recommendation.

Reviewer #1: (No Response)

2. Is the manuscript technically sound, and do the data support the conclusions?

The manuscript must describe a technically sound piece of scientific research with data that supports the conclusions. Experiments must have been conducted rigorously, with appropriate controls, replication, and sample sizes. The conclusions must be drawn appropriately based on the data presented.

Reviewer #1: Yes

3. Has the statistical analysis been performed appropriately and rigorously?

Reviewer #1: Yes

4. Have the authors made all data underlying the findings in their manuscript fully available?

The PLOS Data policy requires authors to make all data underlying the findings described in their manuscript fully available without restriction, with rare exception (please refer to the Data Availability Statement in the manuscript PDF file). The data should be provided as part of the manuscript or its supporting information, or deposited to a public repository. For example, in addition to summary statistics, the data points behind means, medians and variance measures should be available. If there are restrictions on publicly sharing data—e.g. participant privacy or use of data from a third party—those must be specified.

Reviewer #1: No

*Please note that we have uploaded our interview transcripts.*

5. Is the manuscript presented in an intelligible fashion and written in standard English?

PLOS ONE does not copyedit accepted manuscripts, so the language in submitted articles must be clear, correct, and unambiguous. Any typographical or grammatical errors should be corrected at revision, so please note any specific errors here.

Reviewer #1: Yes

6. Review Comments to the Author

Please use the space provided to explain your answers to the questions above. You may also include additional comments for the author, including concerns about dual publication, research ethics, or publication ethics. (Please upload your review as an attachment if it exceeds 20,000 characters)

Reviewer #1: Thank you for your submitted revision of this manuscript.

*Thank you for your ongoing support of our project. We appreciate the conscientiousness of your comments and your careful eye. We have made several edits throughout, which we believe makes the manuscript much stronger. Many thanks.*

There are still some underlying methodological issues that have not been adequately addressed.

In reviewing the transcripts, I have some additional questions/concerns.

It seems that not everyone was asked all of the same questions, particularly JC.

*The interviews were semi-structured. As you see in the transcripts, the questions are open-ended and allow for non-biased responses. While nearly the same questions were asked of all participants, there may be variation. JC was the first interview and several important themes were captured. As we refined the themes, based on emergent ideas, further interviews delved into those themes more deeply. This is the process of grounded theory in qualitative studies.*

Given the sample size, there seem to be a relatively high number of medical directors/other leadership in this cohort. Please address this in the manuscript particularly considering that leadership/local hospital admin was a threat.

*This is a good point. Of the 23, eight had administrative duties. We clarify this in the results with the line, “*Eight (35%) of the participants held leadership positions: regional director (1), medical director (5), assistant medical director (2).”

*In the limitations section, we add the line, “Eight of the participants held leadership positions. This may represent a confounding variable. They may experience extra pressure in their leadership roles or less pressure since their administrative duties lessen their direct-facing patient care. However, their responses resonated with non-leaders with the one difference being that the did not regard leadership as a threat.”*

While age is described in table 1, perhaps it would be more meaningful to use years in practice. It seems that total years in practice was not asked, only years in the current job. Is this correct? If so, is this a potential limitation or co-founder? Years in current job should also be added to Table 1. There are some with many years in the same job but many with relatively few years in the current job.

*While we did ask the participant’s age and total number of years in the present job, we did not ask about total years as an ED physician. We have included this in Table 1. With an average age of 45, most participants were mid-career. While length of time as an ED physician may be helpful for quantitative, survey-based studies, for this qualitative study to explore physician thriving, a broad, representative sample of ED physicians that generally reflect the demographics of ED physicians nationally is helpful here.*

Did this cohort include a pediatrician and IM or FM physician? This should be clarified.

*All were board certified ED physicians. We now note this in the results section.*

Were participants asked about admin hours/month? Clearly there are some non-clinical work hours for the medical directors/admin folks and likely some for the purely clinical folks too. There seemed to be inconsistencies in asking about the number of hours worked.

*In several quantitative studies, increased work hours is associated with burnout. However, in this qualitative study, we explored broad themes of personality types, psycho-social support, and other factors in an open-ended, semi-structured fashion. We were not specifically controlling for work hours. In these interviews, work hours specifically did not emerge as a specific factor, but rather, being socially connected with significant outside interests did. This is the particular strength of qualitative studies and this project in particular. The issue regarding thriving may not be work hours, but rather the quality of time within and outside of work. In a similar study, work hours also did not emerge as a notable theme, but social connection did (JGIM 2021 Dec;36(12):3759-3765. doi: 10.1007/s11606-021-06883-6.)*

The sample seemed to include several individuals that would meet criteria for burnout and/or scored 6 or lower on the satisfaction scale. By your definition (>7), shouldn't these people have been excluded? There were more than 2 individuals who met one of these criteria.

*While these interviews were included in the transcripts, they were excluded from analysis.*

Abstract

Please add the study dates to the abstract

*Done. We have added, “…* from June-September 2020.”

Methods

Appendix A - where is this? It was not provided in the materials to review.

*I uploaded this but apparently it was not in the final pdf. I will make sure it is included this time around.*

Additionally, these questions were a "standardized list". Please clarify. Where was the list derived from? How were these questions decided upon?

My prior comment was misunderstood. As we know that burnout can be transient, it would seem important to have ascertained when these interviews occurred. If most interviews occurred after someone had one day off vs multiple days off vs just worked multiple days in a row, the answers provided in the qualitative survey may have been different among the same individual. There were mention of external (home) stressors by several participants and this should also be noted.

*In general, burnout does not necessarily change dramatically based on a few days off, but tends to be stable over time. Since this project was designed to explore broad themes of physician thriving – and not burnout – the specifics of whether a participant had a day off seems not so relevant here. The questions are designed to be broad, “Overall, how satisfied are you….” “Share with us your views about work-life balance….”*

*Physician thriving is a relatively new concept in the literature. While burnout has been well-studied – indeed, many of us are burned out from burnout – what works has not been well understood or well-described. What makes a physician thrive? We thought it appropriate to ask physicians who were satisfied with life and satisfied with their career (two validated instruments) and also were not burned out (using another validated instrument). The initial questions were derived from review of the literature, namely the work of Tait Shanafelt and others, of which much is theoretical and has not been explored in qualitative studies. Also, as the interviews are semi-structured, the questions are meant to serve as a starting point for a reflective conversation. We believe this is an important strength of the project.*

Results

Please provide rates for gender and ethnicity in the body of the paper as they are presented in the abstract.

*Done.*

The first paragraph is confusing and contradicts itself, please revise. "None of the physicians met criteria for burnout." "Two physicians contacted by the interviewer met criteria for burnout ..."

Unclear if their pre-screen survey was negative and then responses changed at the time of the interview.

*We have clarified this to read, “*There were 25 interviews conducted with emergency physicians across 10 hospitals [Table 1]. Two physicians contacted by the interviewer met criteria for burnout and were not included in the study, leaving 23 for data analysis.”

Based on the methodology, should those with life satisfaction or career satisfaction less than 7 have been excluded?

*This is correct. Two did not meet our inclusion criteria and were excluded from analysis.*

Strengths/Limitations

It should be mentioned that this survey was conducted during COVID. The authors mention in their response letter that rates in the West were low at that time, however, vaccination wasn't yet available and there were still so many clinical unknowns that it must be stated that COVID likely impacted satisfaction. It is the elephant in the room and must be addressed in some way.

As this was a community-based study, it should be specified that these findings may not be generalizable to academic emergency physicians.

Please specify the "diversity of participants" further. In years of practice? Practice location? Seniority? Leadership?

*In the discussion section, we address the COVID-19 pandemic, “*These interviews were conducted during a relative trough in the COVID-19 pandemic at many of the hospitals. We believe this makes these results compelling since these physicians met criteria for thriving and were not burned out during a pandemic. These emergent themes – resilience, social connection, outside interests, with personality types of introversion and extroversion – were associated with career and life satisfaction during a particularly stressful period for the healthcare system.*”*

*In our limitations section, we did have a line, “these findings may not be generalizable to all emergency physicians.” Now, we add the clause, “especially those in academic medicine as this was a community-based study.”*

*In our results section, we do have the line, “Ethnicity and gender were similar to other large studies of emergency physicians [17-19].” We affirm this again in our discussion. Now, in the limitations section, we discuss that 8 of the 23 participants held leadership positions.*

Consider creating a visual representation of the "thriving physician model" from your results.

*We have done so… It looks pretty good!*

7. PLOS authors have the option to publish the peer review history of their article (what does this mean?). If published, this will include your full peer review and any attached files.

If you choose “no”, your identity will remain anonymous but your review may still be made public.

Do you want your identity to be public for this peer review? For information about this choice, including consent withdrawal, please see our Privacy Policy.

Reviewer #1: No

[NOTE: If reviewer comments were submitted as an attachment file, they will be attached to this email and accessible via the submission site. Please log into your account, locate the manuscript record, and check for the action link "View Attachments". If this link does not appear, there are no attachment files.]

While revising your submission, please upload your figure files to the Preflight Analysis and Conversion Engine (PACE) digital diagnostic tool, https://pacev2.apexcovantage.com/. PACE helps ensure that figures meet PLOS requirements. To use PACE, you must first register as a user. Registration is free. Then, login and navigate to the UPLOAD tab, where you will find detailed instructions on how to use the tool. If you encounter any issues or have any questions when using PACE, please email PLOS at figures@plos.org. Please note that Supporting Information files do not need this step.
