# Supplementary material for: Job and life satisfaction among emergency physicians: A qualitative study
Source: PLoS One. 2023 Feb 24;18(2):e0279425. doi: 10.1371/journal.pone.0279425 (PMC9955602; doi:10.1371/journal.pone.0279425)
Supplement: Attachment — Submitted filename: 2022 Nov-Author Response To Reviewers.docx [file pone.0279425.s005.docx]

**Author Response To Reviewers**

Reviewer #1: Thank you for your revisions.

The results section still needs work. Some areas have multiple quotes and others do not. Considering the qualitative nature of the paper, additional quotes are needed to support the themes. The richness of the quotes will amplify the themes and make them more interesting.

*Many thanks for your continued support of our manuscripts.*

*We have included several additional quotes throughout to support our findings. Each domain has 4-6 quotations. This feels about right and is consistent with similar qualitative studies.*

A comment about snowball sampling, it is appropriate for the methodology but does have limitations which should be mentioned. If like finds like and more engaged and extroverted people are responding and passing along to similar people, there is potential for bias in sampling. This should be commented on.

*While snowball sampling has its limits, this was the proper strategy for this project, given the specialized subpopulation (ie. not burned out AND also satisfied with life and their job) (doi: 10.1111/j.1442-2018.2010.00541.x), we do acknowledge the limitations, and include the following line, “While snowball sampling was the appropriate technique for reaching this specialized subpopulation, limitations should be acknowledged. Namely, these participants were a non-random sample. Physicians may have referred participants in their social network, with similar beliefs and ideas. Thus, there may have been themes that were not captured. However, given the diversity of the participants, with ten hospitals represented, and thematic saturation after ten interviews, we believe our sample population represented valid findings.”*
